# Global Analysis of the Evolution and Mechanism of Echinocandin Resistance in *Candida glabrata*

**DOI:** 10.1371/journal.ppat.1002718

**Published:** 2012-05-17

**Authors:** Sheena D. Singh-Babak, Tomas Babak, Stephanie Diezmann, Jessica A. Hill, Jinglin Lucy Xie, Ying-Lien Chen, Susan M. Poutanen, Robert P. Rennie, Joseph Heitman, Leah E. Cowen

**Affiliations:** 1 Department of Molecular Genetics, University of Toronto, Toronto, Ontario, Canada; 2 Department of Biology, Stanford University, Stanford, California, United States of America; 3 Department of Molecular Genetics and Microbiology, Duke University Medical Center, Durham, North Carolina, United States of America; 4 University Health Network/Mount Sinai Hospital, Department of Microbiology, Toronto, Ontario, Canada; 5 Department of Laboratory Medicine and Pathobiology, University of Toronto, Toronto, Ontario, Canada; 6 Department of Medicine, University of Toronto, Toronto, Ontario, Canada; 7 Department of Laboratory Medicine and Pathology, University of Alberta, Edmonton, Alberta, Canada; Carnegie Mellon University, United States of America

## Abstract

The evolution of drug resistance has a profound impact on human health. *Candida glabrata* is a leading human fungal pathogen that can rapidly evolve resistance to echinocandins, which target cell wall biosynthesis and are front-line therapeutics for *Candida* infections. Here, we provide the first global analysis of mutations accompanying the evolution of fungal drug resistance in a human host utilizing a series of *C. glabrata* isolates that evolved echinocandin resistance in a patient treated with the echinocandin caspofungin for recurring bloodstream candidemia. Whole genome sequencing identified a mutation in the drug target, *FKS2*, accompanying a major resistance increase, and 8 additional non-synonymous mutations. The *FKS2-T1987C* mutation was sufficient for echinocandin resistance, and associated with a fitness cost that was mitigated with further evolution, observed *in vitro* and in a murine model of systemic candidemia. A *CDC6-A511G(K171E)* mutation acquired before *FKS2-T1987C(S663P)*, conferred a small resistance increase. Elevated dosage of *CDC55*, which acquired a *C463T(P155S)* mutation after *FKS2-T1987C(S663P)*, ameliorated fitness. To discover strategies to abrogate echinocandin resistance, we focused on the molecular chaperone Hsp90 and downstream effector calcineurin. Genetic or pharmacological compromise of Hsp90 or calcineurin function reduced basal tolerance and resistance. Hsp90 and calcineurin were required for caspofungin-dependent *FKS2* induction, providing a mechanism governing echinocandin resistance. A mitochondrial respiration-defective petite mutant in the series revealed that the petite phenotype does not confer echinocandin resistance, but renders strains refractory to synergy between echinocandins and Hsp90 or calcineurin inhibitors. The kidneys of mice infected with the petite mutant were sterile, while those infected with the *HSP90*-repressible strain had reduced fungal burden. We provide the first global view of mutations accompanying the evolution of fungal drug resistance in a human host, implicate the premier compensatory mutation mitigating the cost of echinocandin resistance, and suggest a new mechanism of echinocandin resistance with broad therapeutic potential.

## Author Summary

The evolution of drug resistance poses a severe threat to human health. *Candida glabrata* is a leading cause of mortality due to fungal infections worldwide. It can rapidly evolve resistance to drugs such as echinocandins, which target the fungal cell wall and are front-line therapeutics for *Candida* infections. We harness whole genome sequencing to provide a global view of mutations that accumulate in *C. glabrata* during the evolution of echinocandin resistance in a human host. Nine non-synonymous mutations were identified, including one in the echinocandin target. A mutation in an additional gene conferred a small resistance increase and another was in a gene whose dosage mitigated the fitness cost of resistance. We further discovered that compromising function of the molecular chaperone Hsp90 abrogates drug resistance and reduces kidney fungal burden in a mouse model of infection. Hsp90 and its downstream effector calcineurin are required for induction of the drug target in response to drug. Thus, we reveal the first global portrait of antifungal resistance mutations that evolve in a human host, identify the first compensatory mutation that mitigates the cost of echinocandin resistance, and suggest a new mechanism of echinocandin resistance that can be exploited to treat life-threatening fungal infections.

## Introduction

The emergence of drug resistance is an evolutionary process with a profound impact on human health. The widespread deployment of antimicrobial agents in medicine and agriculture exerts strong selection for organisms with enhanced capacity to survive and reproduce in the presence of drug, which has led to the rapid emergence of drug resistance in diverse pathogen populations [1]–[4]. The evolution of drug resistance compromises the efficacy of drugs that we depend on critically for a myriad of therapeutic interventions, and has striking economic consequences. The annual cost attributable to the evolution of drug resistance in the United States alone exceeds $33 billion to cover treatment of patients with drug-resistant infections, additional pesticides required to manage resistant pests, and loss of crops to resistant pests [5]. The emergence of drug resistance in fungal pathogens is of particular concern given the increasing incidence of invasive fungal infections, and the limited number of antifungal drugs. Fungi can cause life-threatening infectious disease in immunocompromised hosts, as well as in healthy humans, and the incidence of fungal bloodstream infections has increased by 207% in recent decades [6]–[8]. Fungi are eukaryotes and share close evolutionary relationships with their human hosts, which limits the number of drug targets that can be exploited to selectively kill fungal pathogens yet minimize host toxicity [1], [3]. Even with current treatment options, mortality rates due to invasive fungal infections can reach 50–90% depending on the pathogen and patient population [7], [8], demanding new strategies to prevent the evolution of drug resistance and enhance the efficacy of antifungal drugs.

The evolution of drug resistance is contingent on genetic variability, the ultimate source of which is mutation. One of the most fundamental questions of central importance to predicting and preventing the evolution of drug resistance is which mutations accompany the evolution of drug resistance in the human host. Developments in sequencing technology [9]–[11] now enable this question to be addressed on a genome-wide scale to reveal the identity of mutations that either confer drug resistance in a clinically relevant context or that modify the fitness consequences of resistance mutations. Whole genome sequencing has been applied to bacteria and has revealed principles underpinning the evolution and transmission of drug-resistant pathogens [12], risk factors for the evolution of drug resistance [13], and population dynamics during the evolution of drug resistance *in vitro* [14]. In fungi, changes in genome-wide gene expression and chromosomal alterations that accompany the evolution of drug resistance have been monitored in experimental populations that evolved resistance *in vitro* [15], [16], and targeted sequence and expression analysis of specific genes has been implemented to identify mechanisms of resistance that evolve in the human host [1], [17]. However, a global approach to mapping mutations that underpin the evolution of fungal drug resistance has yet to be achieved.

*Candida glabrata* is a leading fungal pathogens of humans and provides a particularly powerful system for studying the evolution of drug resistance in a human host. *Candida* species are the fourth most common cause of hospital acquired blood-stream infections and are the most prevalent cause of invasive fungal infection worldwide, with mortality rates approaching 50% [7], [18]. *C. glabrata* is now second to *C. albicans* as the most prevalent *Candida* species isolated from clinical specimens [7], [8], [19]. This is due in part to both intrinsic and rapidly acquired resistance of *C. glabrata* to the azoles, which are the most widely used class of antifungal drugs and inhibit the biosynthesis of the key sterol in fungal cell membranes, ergosterol [20]. As a consequence, the echinocandins are the front-line therapeutic agent for *C. glabrata* infections [19]. *C. glabrata* is closely related to the model yeast *Saccharomyces cerevisiae* and is placed within the *Saccharomyces* clade rather than the *Candida* clade to which the leading cause of candidiasis, *C. albicans*, belongs [21]–[23]. It is thought that *C. glabrata* emerged as a human pathogen independently from other *Candida* species. Notably, gene families associated with pathogenicity in *C. albicans* including iron acquisition and host cell adhesion and invasion are absent from *C. glabrata* [24]. *C. glabrata* is an obligate haploid and mating has never been reported, although two mating types, mating type switching, and other mating and meiotic machinery have been described [25]–[27]. To increase genetic diversity *C. glabrata* undergoes chromosomal translocations and variation in gene copy number [28], [29], mechanisms that also contribute to *C. albicans* resistance to azoles [16], [30], [31]. As a haploid, analysis of *C. glabrata* genome sequence is more facile than in diploids such as *C. albicans*, where mitotic recombination and gene conversion can inflate the number of polymorphisms that accrue and obscure the signal of those functionally associated with drug resistance or adaptation to the host.

Compared to the most widely used antifungal drugs in clinical use for the treatment of systemic infections, the azoles, mechanisms of resistance remain more limited for echinocandins. The echinocandins are the only novel class of antifungal drugs approved for clinical use in decades and target the biosynthesis of the key fungal cell wall component, 1,3-β-D-glucan [3], [20], [32]. The 1,3-β-D-glucan synthases are encoded by *FKS1*, *FKS2*, and *FKS3* in *S. cerevisiae*, *C. glabrata*, and *C. albicans*, and require a regulatory subunit encoded by *RHO1* for activity [3], [20], [32], [33]. It is thought that the echinocandins bind to and inhibit the Fks protein; however, the exact mechanism of inhibition remains unknown [33]. Although the echinocandins have been in clinical use since only 2001, there have been numerous reports of *C. glabrata* echinocandin resistance in patients [34]–[37]. Thus far, the only echinocandin resistance mechanism described is mutation in the drug target, Fks, particularly in highly conserved hot spots regions [33], [36]–[39]. Such mutations can reduce echinocandin susceptibility of 1,3-β-D-glucan synthase by 2 to 3 log orders relative to the wild-type enzyme [36]. Additional resistance mechanisms may remain to be described given that Fks mutations have not been identified in some echinocandin-resistant isolates [20], [37], [40], and that isolates with identical Fks mutations exhibit different resistance phenotypes with distinct responses to cellular perturbations [41]. Even with azoles, for which resistance mechanisms have been studied for decades, new resistance mechanisms and modulators of resistance continue to be discovered, expanding the repertoire of strategies employed by fungi to survive drug exposure to include mutation in the drug target, overexpression of multidrug-efflux transporters, and metabolic alterations that minimize drug toxicity [3], [20], [32]. In addition to these canonical resistance mechanisms where mutations in relevant genes confer resistance, there is also an emergent paradigm in which regulators of cellular stress responses are crucial for enabling the evolution and maintenance of drug resistance [3], [20], [32]; while mutations in these regulators have not been identified as a cause of resistance, stress response regulators are key resistance modulators critical for enabling the phenotypic effects of resistance acquired by diverse mechanisms.

Beyond mapping mutations that confer resistance, there is pressing clinical need to elucidate strategies to block the evolution of drug resistance and abrogate resistance once it has evolved. One of the most well studied examples of a protein that governs the emergence and maintenance of fungal drug resistance is the molecular chaperone Hsp90. Hsp90 regulates the folding and function of diverse client proteins, including many signal transducers [42], [43]. In *C. albicans*, compromise of Hsp90 function reduces basal tolerance and resistance of clinical isolates to both the azoles and the echinocandins [41], [44], [45]. Hsp90 enables crucial responses to drug-induced stress by orchestrating signaling through the protein phosphatase calcineurin and the protein kinase C (PKC) cell wall integrity signaling cascade [41], [46]. Hsp90 stabilizes the catalytic subunit of calcineurin and the terminal mitogen-activated protein kinase (MAPK) in the Pkc1 cell wall integrity pathway. Genetic or pharmacological compromise of Hsp90 function can enhance the efficacy of antifungals against *C. albicans* in multiple metazoan models of infection [41], [45]. Notably, Hsp90's role in governing cellular responses to azoles in *C. albicans* is conserved in *S. cerevisiae* [44], [47]. In contrast, Hsp90 and calcineurin play a key role in crucial cellular responses to echinocandins in *C. albicans*, but not in *S. cerevisiae* [41]. Whether Hsp90 influences drug resistance in *C. glabrata* remains entirely unknown. In *C. glabrata*, both calcineurin and PKC signaling have been implicated in basal tolerance to echinocandins [48], [49], though the role of Hsp90 remains unknown as does the impact of any of these regulators on *bona fide* echinocandin resistance.

Here, we provide the first global analysis of mutations accompanying the evolution of fungal drug resistance in a human host. We report on a series of *C. glabrata* isolates that evolved echinocandin resistance in a patient undergoing treatment with the echinocandin caspofungin for recurring *C. glabrata* candidemia over a 10-month period. Whole genome sequencing revealed that a mutation occurred in the gene encoding the drug target, *FKS2*, accompanying a major increase in resistance, as well as 8 other non-synonymous mutations in genes not previously implicated in echinocandin resistance, including *CDC6* and *CDC55*. The *FKS2-T1987C(S663P)* mutation was sufficient to confer echinocandin resistance in a susceptible laboratory strain; however, the mutant allele also imparted a growth defect in clinically relevant conditions using RPMI medium at 37°C. The fitness cost of resistance was mitigated with further evolution, and this trend was also observed in a murine model of disseminated infection. A *CDC6-A511G (K171E)* mutation acquired prior to the *FKS2-T1987C(S663P)* mutation was sufficient to confer a small increase in resistance. Elevated dosage of *CDC55*, which acquired a *C463T(P155S)* mutation after *FKS2-T1987C(S663P)*, ameliorated the fitness cost imparted by the *FKS2* mutation. To uncover mechanisms that abrogate echinocandin resistance, we turned to Hsp90 and found that genetic or pharmacological compromise of *C. glabrata* Hsp90 function reduced basal tolerance and resistance of clinical isolates. Compromising calcineurin function pharmacologically or genetically phenocopied compromising Hsp90 function. Caspofungin induced *FKS2* expression in a manner that depended upon Hsp90 and calcineurin, providing a molecular mechanism by which Hsp90 and calcineurin regulate echinocandin resistance. Furthermore, one of the clinical isolates in the series is a petite mutant based on morphology and inability to respire; although the petite phenotype was not intrinsically involved in echinocandin resistance, it imparted resistance to the combination of echinocandins and inhibitors of Hsp90 or calcineurin. In a mouse model of candidemia, the petite mutant was rapidly cleared and completely avirulent while infection with the *HSP90*-repressible strain yielded a reduced fungal burden compared to wild type. Thus, our results provide the first global view of mutations that accompany the evolution of fungal drug resistance in a human host, implicate the first compensatory mutation that mitigates the cost of echinocandin resistance, and suggest a new molecular mechanism regulating echinocandin resistance, with broad therapeutic potential.

## Results

### The evolution of echinocandin resistance in *C. glabrata* in a human host

While numerous echinocandin-resistant isolates have been recovered from patients [34]–[37], in most cases, there has not been adequate sampling over the course of drug treatment to identify related fungal lineages with which to study the evolution of drug resistance in the human host. The most detailed sampling includes a clinical isolate pre-caspofungin treatment and two isolates taken serially post-caspofungin treatment, however, the mechanism by which resistance evolved was not investigated [50]. In contrast, series of clinical isolates recovered over time from patients undergoing treatment with azoles have proven instrumental for dissecting mechanisms of azole resistance in *C. albicans* [44], [51]–[55]. The more detailed sampling and analysis of the evolution of resistance to azoles likely reflect the fact that azoles have been used clinically for a longer duration.

We report here on a series of *C. glabrata* isolates recovered over a 10-month period from a 45-year old female patient with Crohn's disease who suffered from recurrent *C. glabrata* candidemia and underwent multiple rounds of caspofungin treatment before ultimately succumbing to the infection. A case report on the details of the patient history and therapeutic interventions is provided in the Materials and Methods. Given that multiple blood cultures were negative prior to stopping treatment at any interval and that susceptibility testing was not routinely performed at the time of treatment, caspofungin remained the main therapeutic intervention for candidemia. The 7 blood culture isolates analyzed are labeled alphabetically in the order in which they were recovered, such that isolate A was recovered prior to caspofungin treatment and isolate G was recovered 10 months after recurrent infection and several rounds of caspofungin treatment. The isolates were determined to be related based on molecular typing analysis including pulsed-field gel electrophoresis (PFGE) - karyotype analysis (Figure S1), as well as restriction enzyme PFGE using SfiI (data not shown). Antifungal susceptibility of the 7 isolates in the series was determined for caspofungin, as well as for numerous azoles (fluconazole, ketoconazole, itraconazole, and voriconazole) and for amphotericin B, which binds to ergosterol and disrupts membrane integrity [20], using broth microdilution with RPMI 1640 and following the standard CLSI M27-A3 protocol [56] (Table S1). The major trend observed was an increase in caspofungin resistance over the course of treatment. There were only minimal changes in susceptibility to the other antifungal drugs tested, with the exception of an increase in resistance to azoles that peaked at isolate F and returned to intermediate levels at isolate G. Because the patient was not treated with azoles, the changes in azole susceptibility may be due to mutations that arose in the lineage due to genetic drift rather than selection, or may reflect additional phenotypic consequences of mutations associated with echinocandin resistance, mutations associated with adaptation to the bloodstream, or mutations that were not directly selected for but simply hitch-hiked along with mutations under selection in this predominantly clonal system.

### Whole genome sequencing reveals 9 non-synonymous mutations between early clinical isolate A and late clinical isolate G

The genomic spectrum of resistance mutations that emerge during the evolution of a fungal pathogen in its human host can be addressed for the first time using next-generation sequencing technology and the series of *C. glabrata* clinical isolates we describe here. As a haploid, genome analysis of *C. glabrata* is simplified relative to diploids such as *C. albicans*, where mitotic recombination and gene conversion can amplify the number of polymorphisms observed between early and late isolates (changes from heterozygous to homozygous states are also classified as nucleotide changes) and thereby hinder functional analysis of mutations conferring resistance. Further, *C. glabrata* shares a recent common ancestor with the model yeast *S. cerevisiae* with a large number of orthologs between the two species, facilitating bioinformatic analysis [21], [22]. Given the clinical importance of echinocandin resistance, and that to date resistance has only been attributed to mutations in the echinocandin target [33], [36]–[39], with evidence that additional resistance determinants and modulators may remain to be discovered [20], [37], [40], we sought to identify the mutations that accompany the evolution of echinocandin resistance in the human host on a genome-wide scale.

**Figure 1:**
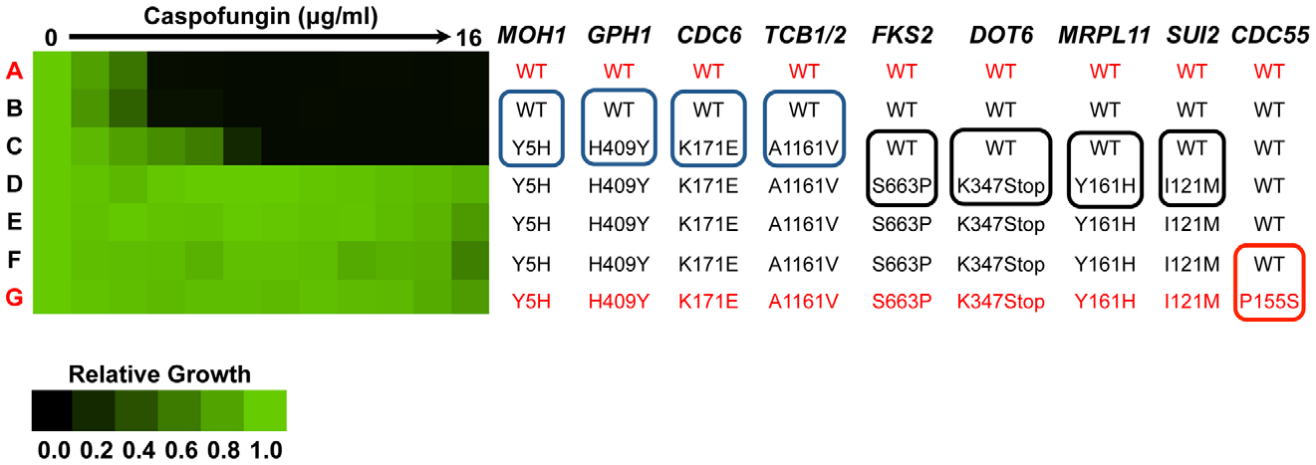
Non-synonymous mutations that accumulate in *C. glabrata* during the evolution of echinocandin resistance in a human host. *C. glabrata* clinical isolates display a step-wise increase in caspofungin resistance in a Minimum Inhibitory Concentration (MIC) assay. Isolates are arranged in the same order as they were recovered from the patient, where isolate A was recovered pre-treatment and isolate G was recovered after multiple rounds of caspofungin treatment. Assays were performed in RPMI medium with 2% glucose at 30°C for 72 hours. Optical densities were averaged for duplicate measurements and normalized relative to caspofungin-free controls (see color bar). The nine non-synonymous mutations identified in isolate G compared to isolate A using whole genome sequencing (marked in red) were mapped across isolates B to F using Sanger sequencing. Mutations in *MOH1*, *GPH1*, *CDC6*, and *TCB1/2* occur between isolates B and C, which correspond to a small increase in echinocandin resistance, outlined in blue boxes. Mutations in *DOT6*, *MRPL11*, *FKS2*, and *SUI2* correspond to a sharp increase in echinocandin resistance, outlined in black boxes.

We performed whole genome sequencing of the *C. glabrata* isolate recovered prior to caspofungin treatment (isolate A) and the last isolate recovered after multiple rounds of treatment (isolate G) using the Illumina Genome Analyzer II platform. We obtained 5.1 and 3.8 million 76 base pair single-end reads for isolate A and isolate G, respectively, resulting in 22 to 30× genome coverage. Reads were aligned against the reference genome sequence of CBS138 [57]. Single nucleotide variants were identified using a machine learning approach. A total of 45,797 single nucleotide variants were identified between late clinical isolate G and CBS138. Of these single nucleotide variants, 39,146 had sufficient sequencing depth in isolate A to be reliably assigned. Overall, 26 single nucleotide variants were uncovered between isolate A and G, with only 17 of these within open reading frames and only 9 resulting in non-synonymous changes (Table 1). Although the 9 mutations that were not within open reading frames (Table 2) could include mutations that affect regulation of genes important for drug resistance, we focused our analysis on mutations within open reading frames given that silent mutations can more readily be distinguished from those with functional consequences in coding sequences and given their potential impact on gene product function. All 9 non-synonymous changes were verified and then mapped across isolates B to F using Sanger sequencing to determine when each mutation arose in the series (Figure 1). Genes are named based on homology to *S. cerevisiae* genes [57].

**Table 1:** Whole genome sequencing reveals 17 single nucleotide variants (SNVs) within open reading frames between early clinical isolate A and late clinical isolate G.

Gene	*S. cerevisiae* Homolog	A Codon	G Codon	A Amino Acid	G Amino Acid	Codon Position
CAGL0F04631g	*MOH1*	TAC	CAC	Y	H	5
CAGL0F04895g	*GPH1*	CAC	TAC	H	Y	409
CAGL0K00605g	*CDC6*	AAA	GAA	K	E	171
CAGL0J08591g	*TCB1/2*	GCA	GTA	A	V	1161
CAGL0K04037g	*FKS2*	TCT	CCT	S	P	663
CAGL0A04257g	*DOT6*	AAA	TAA	K	Stop	347
CAGL0J09724g	*MRPL11*	TAC	CAC	Y	H	161
CAGL0B03795g	*SUI2*	ATC	ATG	I	M	121
CAGL0L06182g	*CDC55*	CCA	TCA	P	S	155
CAGL0A00517g	*PMC1*	GGT	GGC	G	G	241
CAGL0B04279g	*RKM3*	GGT	GGC	G	G	49
CAGL0C02211g	*UTR2*	TTC	TTT	F	F	11
CAGL0D03344g	*UBR2*	TCG	TCA	S	S	1681
CAGL0I02288g	*CDC23*	TGC	TGT	C	C	42
CAGL0J07040g	*GDE1*	GTA	GTC	V	V	495
CAGL0M04653g	*PEP3*	TTA	TTG	L	L	633
CAGL0M13541g	*TDA1*	ATC	ATT	I	I	83

Whole genome sequencing was performed on clinical isolates A and G using the Illumina GAII platform. All high-confidence SNVs located within predicted open reading frames identified between the early clinical isolate A and late clinical isolate G are listed. Genome coverage of 22 to 30× was obtained, with a total of 45,797 SNVs identified between isolate A and the reference CSB138 strain.

**Table 2:** Whole genome sequencing reveals 9 single nucleotide variants (SNVs) that lie outside predicted open reading frames between early clinical isolate A and late clinical isolate G.

(Chromosome) Coordinate	Substitution	Most Proximal Gene(s)	Mutation Position	*S. cerevisiae* Homolog
(Cagl0L) 63245	C to G	CAGL0L00539g	803 bp downstream	*ADD37*
(Cagl0M) 1164711	A to T	CAGL0M11704g (*AHP1*)	608 bp upstream	*AHP1*
(Cagl0J) 162944	A to G	**1.** CAGL0J01774g	1864 bp downstream	N/A
		**2.** CAGL0J01727g	2163 bp upstream	N/A
(Cagl0L) 1294054	C to A	**1.** CAGL0L12034g	243 bp upstream	*ECM32*
		**2.** CAGL0L12012g	480 bp upstream	*TMT1*
(Cagl0J) 46946	T to C	**1.** CAGL0J00539g (*SLT2*)	149 bp upstream	*SLT2*
		**2.** CAGL0J00517g	572 bp downstream	*RRM3*
(Cagl0F) 705090	A to G	CAGL0F07249g	486 bp upstream	*TAF6*
(Cagl0D) 91734	T to C	CAGL0D00704g	316 bp downstream	*YET3*
(Cagl0C) 90199	T to C	CAGL0C00847g (*EPA8*)	1696 bp downstream	*FLO10*
(Cagl0K) 573053	G to T	CAGL0K05841g (*HAP1*)	91 bp upstream	*HAP1*

All high-confidence SNVs that were found outside of predicted open reading frames between early clinical isolate A and late clinical isolate G are listed. Their genome coordinates, the base pair substitution, the most proximal gene(s), the position of the mutation relative to that gene, as well as the gene homolog in *S. cerevisiae* are listed.

Mutations in the *MOH1, GPH1, CDC6,* and *TCB1/2* genes accompanied the first modest increase in resistance in the series at isolate C (Figure 1). The function of *MOH1* in *S. cerevisiae* is largely unknown except that it is required for survival in stationary phase [58], [59], and it was found to genetically interact with Hsp90 in a genome-wide chemical-genetic screen [60]. Expression of *C. albicans MOH1* is induced by alpha pheromone in filament-inducing Spider medium, by weak acid stress via Mnl1, and in biofilm conditions [61]–[63]. *GPH1* encodes a glycogen phosphorylase regulated by the high osmolarity glycerol (HOG) mitogen-activated protein (MAP) kinase pathway in *S. cerevisiae* [64], [65], and is induced upon fluconazole treatment in *C. albicans* [66]. The *CDC6* gene product is involved in DNA replication initiation by forming and maintaining the pre-replicative complex and serving as a loading factor for the Mcm2–7 proteins onto chromatin [67], [68]. *TCB1* and *TCB2* encode proteins containing both calcium and lipid binding domains and appear to be involved in membrane trafficking in *S. cerevisiae* [69], [70]. None of these genes or proteins have been previously implicated in echinocandin resistance.

Additional mutations emerged accompanying the subsequent major increase in echinocandin resistance, and in the last isolate of the series. Mutations in *FKS2*, *DOT6*, *MRPL11*, and *SUI2* accompanied the largest increase in echinocandin resistance at isolate D (Figure 1). Of these genes, only *FKS2* has been implicated in echinocandin resistance. *FKS2* encodes the catalytic subunit of 1,3-β-D-glucan synthase, the target of the echinocandins, and the Fks2 S663P mutation identified is in mutational hot spot 1 and has been found in other echinocandin-resistant *C. glabrata* clinical isolates [36], [37], [71]. Dot6 is a subunit of the RPD3L histone deacetylase complex in *S. cerevisiae* and is involved in both pseudohyphal morphogenesis as well as silencing at telomeres [72]–[74]. Mrpl11 is a mitochondrial protein and part of the large ribosomal subunit [75], [76]. *SUI2* plays a role in translation initiation and encodes the alpha subunit of the translation initiation factor eIF2 in *S. cerevisiae* [77]. Finally, a non-synonymous mutation occurred in *CDC55* in the latest clinical isolate G, despite no further increase in echinocandin resistance (Figure 1). *CDC55* encodes the regulatory B subunit of protein phosphatase 2A and has a number of functions, including roles in spindle assembly during meiosis, mitotic exit, pseudohyphal morphogenesis, and chromosome disjunction [78]–[81].

Thus, in addition to a mutation in the known echinocandin target, whole genome sequencing revealed the acquisition of 8 additional non-synonymous mutations in 8 genes not previously implicated in echinocandin resistance or adaptation to host conditions during the evolution of echinocandin resistance in a human host. The genome sequence analysis further confirms clonality of the lineage given the very limited number of single nucleotide variants genome-wide compared to large number observed between the late clinical isolate G and the reference genome CBS138. Further, that each of the mutations identified persisted throughout the lineage once it emerged suggests that there may have been strong selective sweeps in the population such that polymorphisms rapidly reached near fixation.

### The *FKS2* T1987C (S663P) mutation confers echinocandin resistance and imparts a fitness deficit in a *C. glabrata* laboratory strain

**Figure 2:**
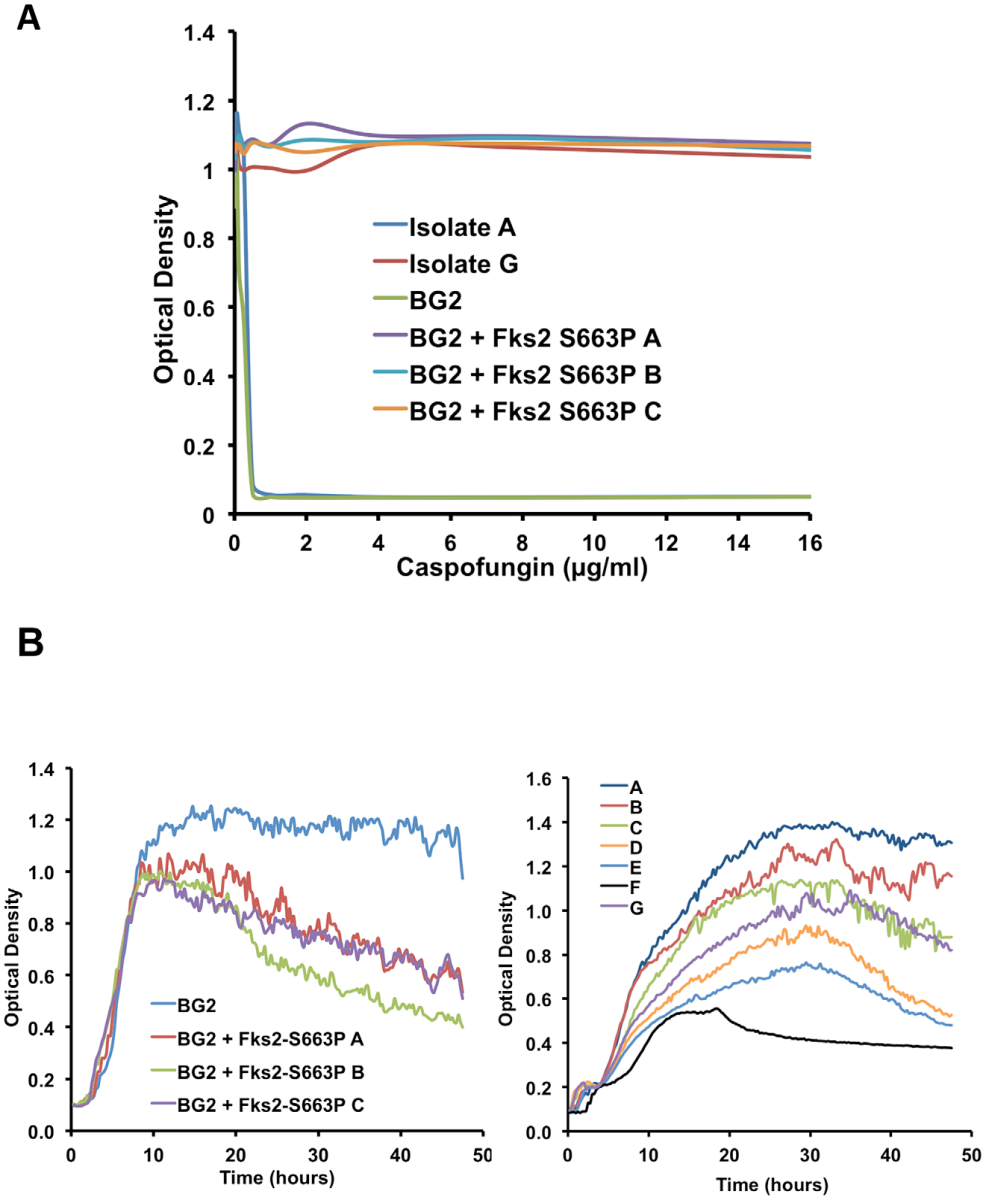
The *FKS2* T1987C (Fks2 S663P) mutation is sufficient for echinocandin resistance in a *C. glabrata* laboratory strain and imparts a fitness cost in the absence of the drug. (**A**) The *FKS2* T1987C (Fks2 S663P) mutation uncovered in caspofungin-resistant clinical isolate G was introduced into a sensitive laboratory *C. glabrata* strain BG2 via single-stranded DNA containing a silent marker. Resistance profiles of three independent sequence-verified transformants were tested via MIC. MIC assays were performed in Figure 1 and plotted graphically as caspofungin concentration (µg/ml) versus optical density at 600 nm. (**B**) Growth kinetics of the BG2 laboratory strain of *C. glabrata* and three independent transformants harbouring a mutant *FKS2* T1987C allele (left panel) and of the *C. glabrata* clinical isolate series (right panel). Growth curves were performed in RPMI medium at 37°C with orbital shaking, with measurements taken every 15 minutes for 48 hours.

To determine which of the mutations identified by whole genome sequencing contributes to echinocandin resistance we first turned to the most likely candidate resistance gene, *FKS2*. The *FKS2* mutation that emerged in isolate D and was maintained throughout the rest of the series (T1987C) results in substitution of a serine to proline at amino acid 663 in the target of the echinocandins. This *FKS2* T1987C (S663P) mutation has been previously associated with high levels of caspofungin resistance in *C. glabrata* clinical isolates [36], [37]. *In vitro* biochemical studies established that this mutant Fks2 enzyme displays reduced sensitivity to inhibition by echinocandins, as indicated by a higher kinetic inhibition parameter, as well as decreased catalytic capacity, and reduced enzyme velocity, compared to the wild-type enzyme; notably the binding affinity of the mutant enzyme for echinocandins remains unchanged [36]. While there is an association of *FKS* mutations with resistance, and biochemical data support the resistance mechanism, it has only been conclusively demonstrated that such mutations are sufficient to confer echinocandin resistance in *S. cerevisiae* [82], [83]. To test whether Fks2 S663P is sufficient to confer echinocandin resistance, we introduced the T1987C mutation into the sensitive laboratory strain BG2 using a strategy involving single-stranded DNA containing the mutation and a silent marker, followed by selection of transformants on medium containing caspofungin. No echinocandin-resistant colonies were obtained following control transformations with single-stranded DNA containing the equivalent wild-type sequence or with a water control, while many resistant colonies were obtained with the sequence containing the T1987C mutation. We assessed resistance of four sequence-verified transformants that harboured both the T1987C and silent mutation via minimum inhibitory concentration (MIC) assays. The transformants displayed resistance similar to clinical isolate G (Figure 2A). Thus, Fks2 S663P is sufficient to confer echinocandin resistance in a susceptible laboratory strain of *C. glabrata*.

Given that specific Fks amino acid substitutions that decrease sensitivity of the 1,3-β-D-glucan synthase enzyme to echinocandins also reduce the enzyme catalytic capacity, the Fks2 S663P mutation may confer a fitness cost in terms of reduced growth or viability in the absence of the drug [36], [39]. To determine if the *FKS2* mutations compromises fitness, we monitored growth kinetics of the lab strain BG2 and the progeny harbouring the Fks2 S663P substitution. In clinically relevant conditions, RPMI at 37°C, we found that strains harbouring the Fks2 S663P substitution displayed significantly reduced fitness relative to the parental wild-type laboratory strain BG2 (*P*<0.01, area under the curve followed by ANOVA, Bonferroni's Multiple Comparison Test, Figure 2B, left panel). Furthermore, we observed a significant reduction in fitness between isolate A and isolate D (*P*<0.001), which is rescued to some extent in isolate G (*P*<0.01, Figure 2B, right panel). Thus, the *FKS2* mutation that emerged in clinical isolate D is sufficient to confer a high level of caspofungin resistance equivalent to that observed in the late isolate G, however, it is also associated with a cost in terms of reduced fitness in the absence of the drug.

### The fitness cost associated with the *FKS2* T1987C (S663P) mutation is mitigated by increased dosage of *CDC55*

**Figure 3:**
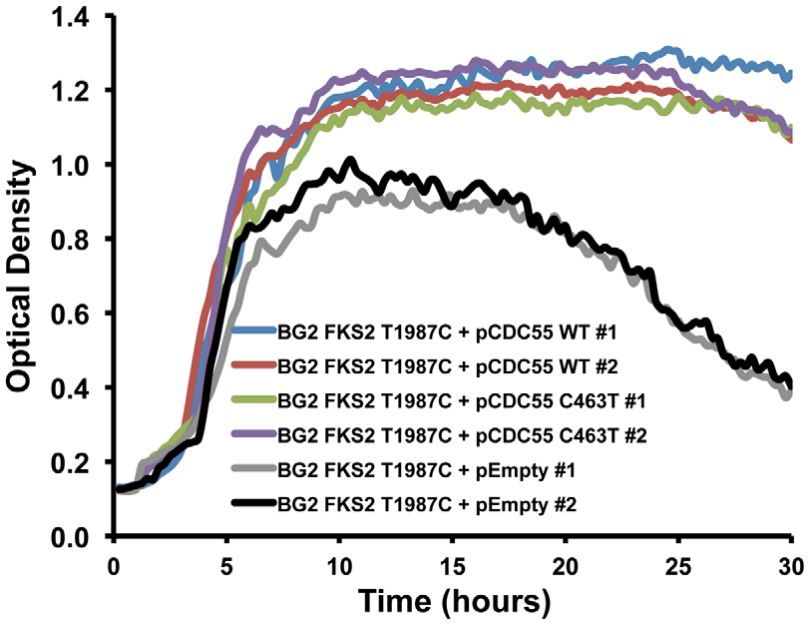
Increased dosage of *CDC55* compensates for the fitness cost associated with the echinocandin resistance mutation *FKS2* T1987C (Fks2 S663P) in *C. glabrata*. Either the wild-type (WT) *CDC55* allele of clinical isolate A or the *CDC55* C463T (Cdc55 P155S) allele of the late resistant clinical isolate G was expressed on a plasmid under the control of its native promoter in a *C. glabrata* laboratory strain harbouring the echinocandin resistance mutation *FKS2* T1987C (Fks2 S663P). Growth kinetics of two independent transformants harbouring the *FKS2* T1987C mutant allele and either the *CDC55* C463T or *CDC55* WT ectopically expressed allele, as well as a control strain harbouring an empty vector, were assessed. Growth curves were performed in RPMI medium at 37°C with orbital shaking, with measurements taken every 15 minutes for 48 hours.

Compensatory mutations that mitigate the cost of drug resistance have been well established in bacterial systems [84]–[87], but remain enigmatic in fungi. Given the fitness cost associated with echinocandin resistance due to mutation in the drug target observed in this study, and in others [88], one would anticipate selection to favour the emergence of compensatory mutations that mitigate the fitness cost of the resistance mutation. The series of *C. glabrata* isolates studied here provide the ideal opportunity to identify compensatory mutations given that a single non-synonymous mutation occurs between isolate D and G, *CDC55* C463T (P155S), associated with an increase in fitness (Figure 1). The *CDC55 C463T* allele from late clinical isolate G was cloned into a plasmid under the control of its native promoter, as was the *CDC55* allele from the early clinical isolate A, and introduced into the BG2 laboratory strain harbouring the *FKS2* T1987C mutation. Monitoring growth kinetics of two independent transformants harbouring each of the *CDC55* plasmids relative to two transformants with the empty vector control demonstrates that the additional copy of *CDC55* ameliorates fitness (P<0.01, ANOVA, Bonferroni Multiple Comparison Test, Figure 3). This suggests that perhaps the *CDC55 C463T* mutation might be a gain-of-function mutation, such that increased fitness could be achieved either by mutation causing increased Cdc55 activity or by increased dosage of *CDC55*. These results identify the premier genetic alteration that mitigates the fitness cost of echinocandin resistance.

### A mutation in *CDC6* confers a small increase in echinocandin resistance

Despite the fact that the *FKS2* T1987C mutation was sufficient to impart the full level of caspofungin resistance of isolate G on an otherwise susceptible laboratory strain (Figure 2A), there is evidence for additional mutations affecting resistance in the evolved lineage. The initial small increase in echinocandin resistance observed at isolate C (Figure 1) occurred prior to the *FKS2* mutation and thus other mutations identified at this early transition may be responsible for this increase in resistance. Notably, isolate C showed a fitness defect in the absence of drug (Figure 2B), suggesting that the mutations imparting this small increase in resistance are also costly. Additional mutations that accumulated could mitigate the fitness cost of resistance mutations.

**Figure 4:**
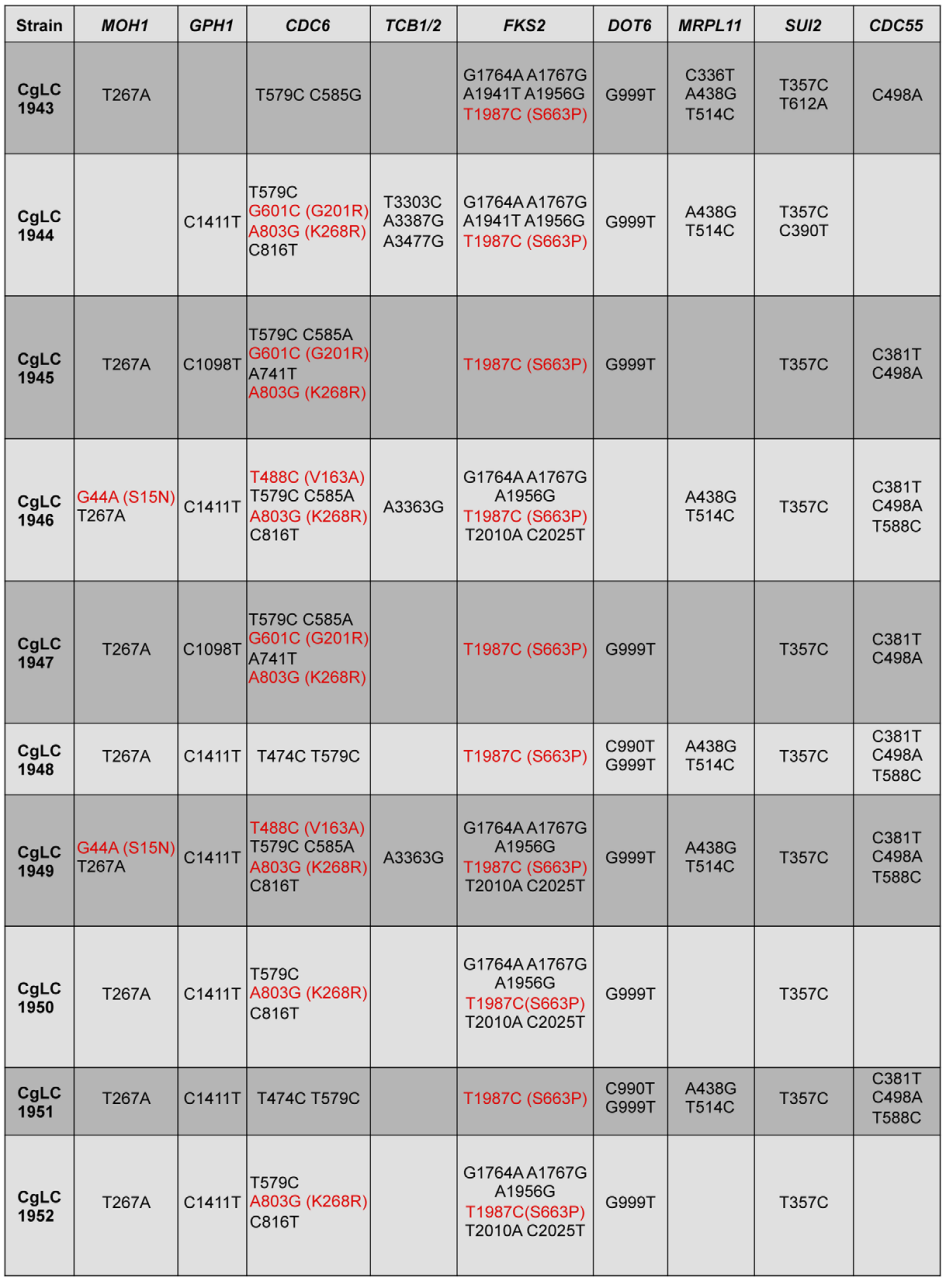
Polymorphisms in *MOH1* and *CDC6* are prevalent in *C. glabrata* clinical isolates with *FKS2-*mediated echinocandin resistance. Genomic regions harbouring the 9 non-synonymous mutations uncovered in isolate G relative to isolate A were sequenced by Sanger sequencing in 10 unrelated *C. glabrata* echinocandin-resistant clinical isolates containing the resistance mutation *FKS2* T1987C/Fks2 S663P. Synonymous mutations are in black font; non-synonymous mutations are in red with amino acid mutations listed in parentheses. Sequences were compared to both the reference CBS138 sequence as well as a control sample sequenced from the laboratory isolate BG2 (CgLC1272).

**Figure 5:**
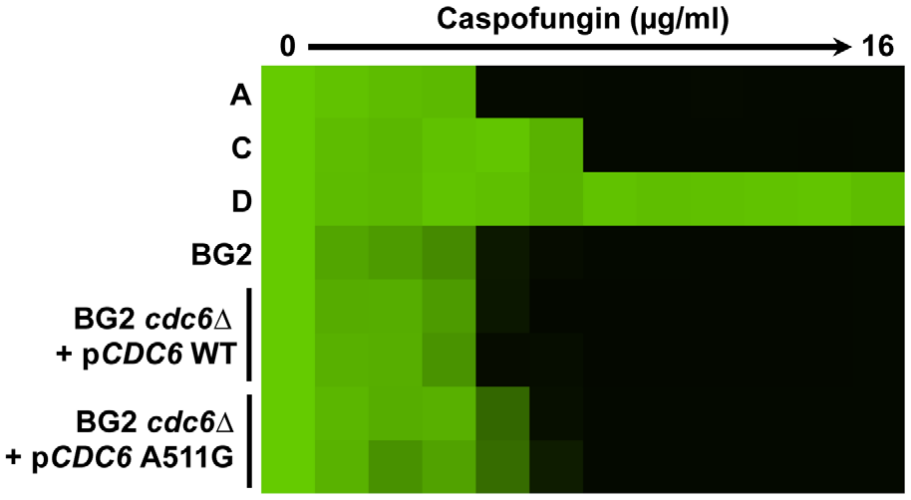
The *CDC6* A511G (Cdc6 K171E) mutation uncovered in clinical isolate C imparts a small increase in echinocandin resistance in *C. glabrata*. The mutant allele of *CDC6* that was identified in clinical isolate C and accompanied a small increase in echinocandin resistance, or a wild-type allele of *CDC6* cloned from the early sensitive isolate A, was expressed on a plasmid under the control of its native promoter and introduced as the only copy of *CDC6* in a laboratory strain of *C. glabrata*. Caspofungin resistance of two independent transformants of strains harbouring the mutant or wild-type *CDC6* allele was assessed using an MIC assay. Assays were performed in synthetic defined medium at 30°C for 72 hours. Data was analyzed as in Figure 1.

To prioritize mutations for functional analysis, we addressed the prevalence of mutations in any of the 8 genes found to harbour mutations in our whole genome sequence analysis in addition to *FKS2* in other *C. glabrata* echinocandin-resistant mutants. To do so, we obtained 10 additional unrelated *C. glabrata* clinical isolates harbouring the Fks2 S663P mutation [71], and sequenced across the 8 genomic regions that were mutated in clinical isolate G via Sanger sequencing. We discovered non-synonymous changes in *MOH1* in two out of the 10 clinical isolates sequenced and non-synonymous changes in *CDC6* in 7 out of 10 of the clinical isolates (Figure 4). Given the prevalence of polymorphisms in *CDC6* and *MOH1* among these echinocandin-resistant clinical isolates, we prioritized these genes for functional analysis. We cloned plasmids with the gene sequences found in isolate A or in isolate G, and expressed these in a susceptible laboratory strain. While the *MOH1-T13C(Y5H)* allele did not confer any increase in echinocandin resistance (data not shown), the *CDC6-A511G(K171E)* allele did confer a small increase in resistance (Figure 5). Thus, we establish a novel mutation in *CDC6* that contributes to reduced echinocandin susceptibility in *C. glabrata* clinical isolates.

### Hsp90 plays a critical role in echinocandin resistance of *C. glabrata* clinical isolates

Given the importance of echinocandins in the therapeutic front line against *C. glabrata* infections and the acute problem imposed by the evolution of echinocandin resistance, there is a pressing need to discover strategies to abrogate drug resistance. We focused on Hsp90 due to its critical role in enabling basal tolerance and acquired antifungal drug resistance in pathogenic fungi such as *C. albicans* and the most lethal mould *Aspergillus fumigatus* [41], [44], [45], [47]. In the context of the echinocandins, *C. albicans* Hsp90 orchestrates crucial cellular responses to survive echinocandin exposure by stabilizing the catalytic subunit of the protein phosphatase calcineurin, while in *S. cerevisiae* Hsp90 and calcineurin do not modulate echinocandin susceptibility under any of the standard conditions tested [41]. To date, no studies have examined the consequences of pharmacological or genetic compromise of Hsp90 function on cellular responses to echinocandins in *C. glabrata*, and thus whether this pathogen shows resistance circuitry more akin to the pathogen *C. albicans* or its closer relative *S. cerevisiae* remains unknown.

**Figure 6:**
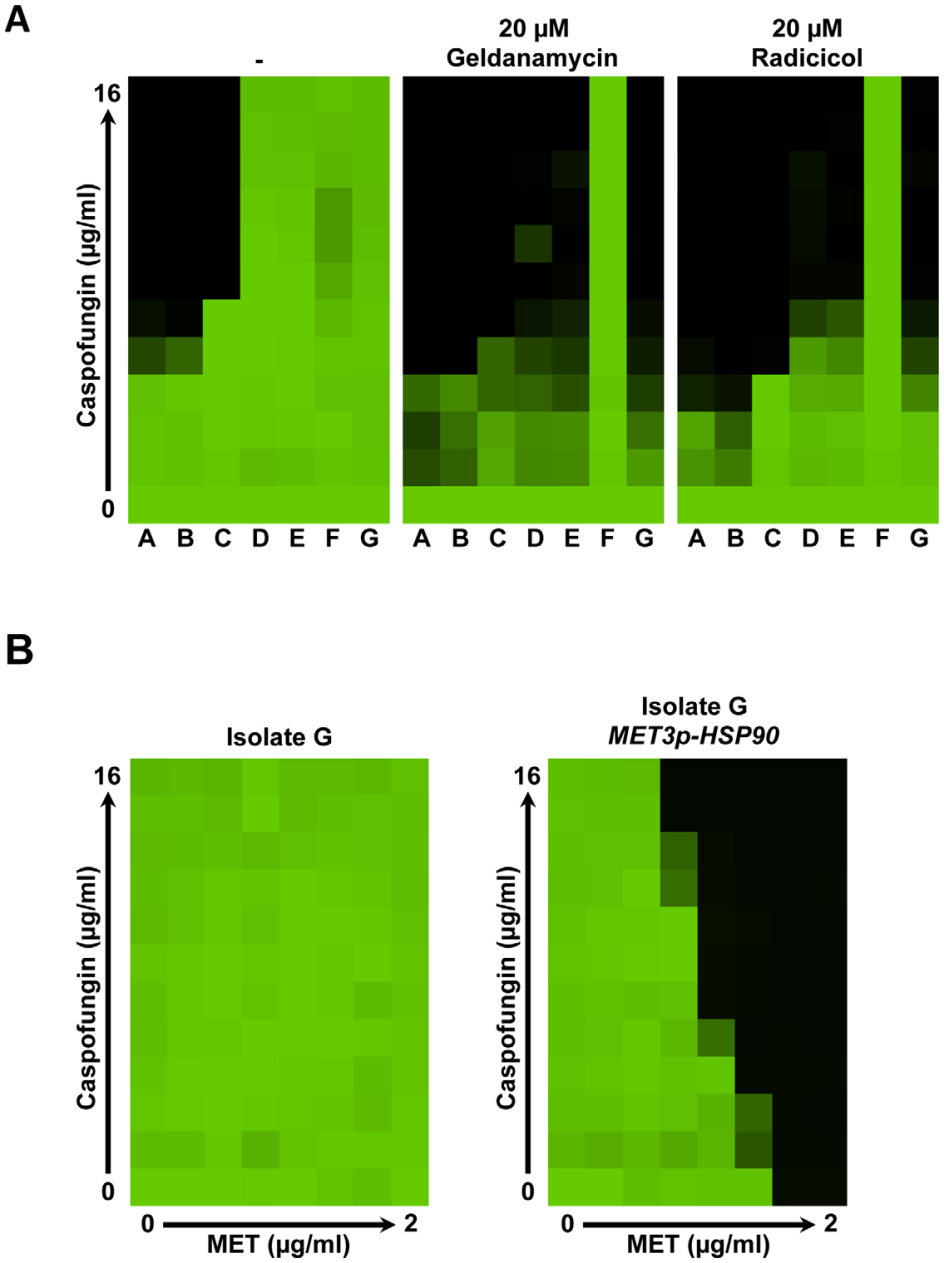
Hsp90 plays a critical role in echinocandin resistance of *C. glabrata* clinical isolates. (**A**) Pharmacological inhibition of Hsp90 with geldanamycin or radicicol reduces caspofungin resistance of *C. glabrata* clinical isolates in an MIC assay. Isolates are arranged in the same order as they were recovered from a patient who was on caspofungin treatment, where isolate A was recovered pre-treatment and isolate G was recovered after multiple rounds of caspofungin treatment. Assays were done in synthetic defined medium at 30°C for 72 hours. Data was analyzed as in Figure 1. (**B**) Genetic depletion of Hsp90 confers hypersusceptibility to caspofungin. Growth of the late, echinocandin-resistant clinical isolate G and its derivative in which *HSP90* expression is driven by the methionine-repressible *MET3* promoter was assessed in a checkerboard format with a gradient of caspofungin and a gradient of methionione. Methionine had no impact on caspofungin resistance of isolate G, but increased the susceptibility of the *MET3p-HSP90* strain in a concentration-dependent manner.

We first implemented a pharmacological approach to determine if inhibition of Hsp90 modulates echinocandin resistance of *C. glabrata*. We monitored growth across a gradient of the widely used echinocandin caspofungin relative to a drug-free growth control in the presence or absence of the two structurally unrelated Hsp90 inhibitors, geldanamycin and radicicol, that bind to the adenosine triphosphate (ATP) binding pocket of Hsp90 and thereby compromise ATP-dependent chaperone function [89], [90]. In the absence of geldanamycin, there was a small increase in caspofungin resistance between isolate B and isolate C and a large increase in resistance between isolate C and isolate D (Figures 1 and 6A). Pharmacological inhibition of Hsp90 with geldanamycin or radicicol decreased tolerance of the early clinical isolates A, B, and C, and reduced resistance of the late clinical isolates D, E, and G (Figure 6A). Synergy between caspofungin and geldanamycin was also observed in RPMI, a medium used for clinical susceptibility testing (Figure S2). Notably, isolate F was refractory to the synergy between caspofungin and Hsp90 inhibitors, as discussed in more detail below.

Next, we validated our pharmacological findings genetically. We engineered a strain of *C. glabrata* in which the only *HSP90* allele was expressed under the control of the *MET3* promoter, which is repressed in the presence of methionine and/or cysteine. We monitored the impact of a gradient of methionine concentrations on growth across a gradient of caspofungin concentrations for both isolate G and its derivative in which *HSP90* expression is driven by the *MET3* promoter. Methionine had no impact on caspofungin resistance of isolate G (Figure 6B). In contrast, methionine reduced echinocandin resistance of the *MET3p-HSP90* derivative of isolate G in a dose-dependent manner, providing genetic validation of the importance of Hsp90 for echinocandin resistance (Figure 6B). The highest concentrations of methionine tested (≥1 µg/ml) blocked growth of the *MET3p-HSP90* strain, consistent with Hsp90's essentiality in all eukaryotes tested. Thus, targeting Hsp90 provides the first and much-needed strategy to abrogate echinocandin resistance of *C. glabrata*.

### Compromising calcineurin function abrogates echinocandin resistance of *C. glabrata* clinical isolates

Hsp90 enables resistance to both the azoles and echinocandins, in large part via the protein phosphatase calcineurin in *C. albicans* [3], [41], [44], [47]. Hsp90 stabilizes the catalytic subunit of calcineurin in both *S. cerevisiae* and *C. albicans* [41], [91], thereby enabling calcineurin-dependent responses to drug-induced cellular stress [3], [92]. While calcineurin has been implicated in basal tolerance to echinocandins in *C. glabrata* [49], whether calcineurin affects *bona fide* resistance remains unknown.

**Figure 7:**
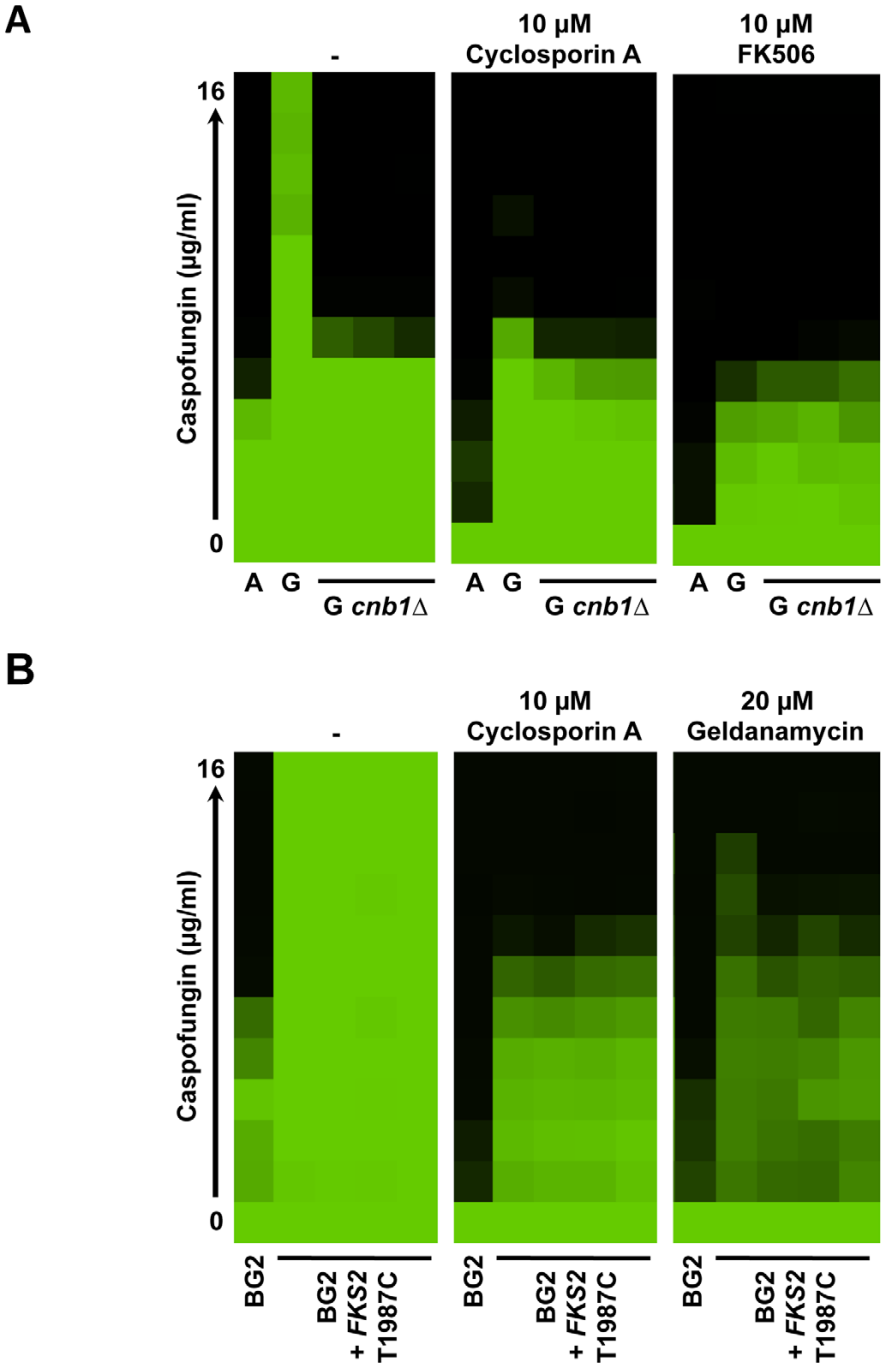
Calcineurin is a key-mediator of Hsp90-dependent echinocandin resistance of *C. glabrata*. (**A**) Genetic or pharmacological compromise of calcineurin function reduces echinocandin resistance of the clinical isolates. Deletion of the regulatory subunit of calcineurin, *CNB1*, required for calcineurin function reduces caspofungin resistance in resistant clinical isolates. Pharmacological inhibition of calcineurin with either cyclosporin A or FK506 also reduces caspofungin resistance. The assay was performed and analyzed as in Figure 1. (**B**) Resistance of the laboratory strain BG2 harbouring the T1987C *FKS2* mutant allele is reduced upon inhibition of calcineurin with cyclosporin A or inhibition of Hsp90 with geldanamycin. Assays were performed as in part (A) and data was analyzed as in Figure 1.

We used both pharmacological and genetic approaches to determine if calcineurin is a key mediator of Hsp90-dependent echinocandin resistance in *C. glabrata*. First, we took a pharmacological approach and assessed growth across a gradient of caspofungin concentrations in the presence or absence of two structurally unrelated calcineurin inhibitors, cyclosporin A and FK506. Cyclosporin A and FK506 inhibit calcineurin function in different ways. Cyclosporin A binds to cyclophilin A, a peptidyl-prolyl cis-trans isomerase, and it is this drug-protein complex that inhibits calcineurin function [93]. FK506 binds a structurally unrelated peptidyl-prolyl cis-trans isomerase, FKBP12, and this distinct drug-protein complex also inhibits calcineurin function [93]. We used concentrations of each calcineurin inhibitor that abolished echinocandin resistance in *C. albicans* but did not inhibit growth on their own [41]. We found that inhibition of calcineurin with cyclosporin A or FK506 reduced caspofungin resistance of the late resistant *C. glabrata* clinical isolate G (Figure 7A). Next, we validated our pharmacological findings genetically by deletion of the regulatory subunit of calcineurin, encoded by *CNB1*, which is required for calcineurin function. Loss of calcineurin function reduced resistance of clinical isolate G in three independent mutants (Figure 7A). Thus, calcineurin is a key mediator of Hsp90-dependent echinocandin resistance in *C. glabrata*.

Since the clinical isolate G harbours multiple mutations relative to its echinocandin-susceptible counterpart, we next specifically assessed the dependence of Fks2-mediated echinocandin resistance on Hsp90 and calcineurin. Pharmacological inhibition of Hsp90 with geldanamycin, or calcineurin with cyclosporin A reduced echinocandin resistance of the laboratory strain harbouring the *FKS2* T1987C allele (Figure 7B), confirming that both Hsp90 and calcineurin are required for echinocandin resistance acquired by mutation of the drug target and providing the first circuitry that governs echinocandin resistance in this pathogen.

### Caspofungin induces *FKS2* expression in a calcineurin- and Hsp90-dependent manner in *C. glabrata*

The specific mechanism by which calcineurin governs echinocandin resistance remains unknown in any system. In *S. cerevisiae*, *FKS2* expression is induced during high temperature growth via calcineurin, and deletion of both *FKS1* and *FKS2* is synthetically lethal [94]–[97]. Based on these findings, we propose a model in which calcineurin regulates echinocandin resistance in *C. glabrata* by controlling expression of the resistance determinant *FKS2*. Given the functional dependence of calcineurin on Hsp90 in other systems [41], [44], [91], one might expect Hsp90 to also influence expression of *FKS2*. According to our model, inhibition of calcineurin or Hsp90 would compromise expression of the echinocandin-resistant 1,3-β-D-glucan synthase and reduce resistance. Compromising calcineurin or Hsp90 function would also reduce basal tolerance of susceptible strains by reducing *FKS2* expression, thereby decreasing the cellular pool of 1,3-β-D-glucan synthase and enhancing susceptibility to a given concentration of echinocandin.

**Figure 8:**
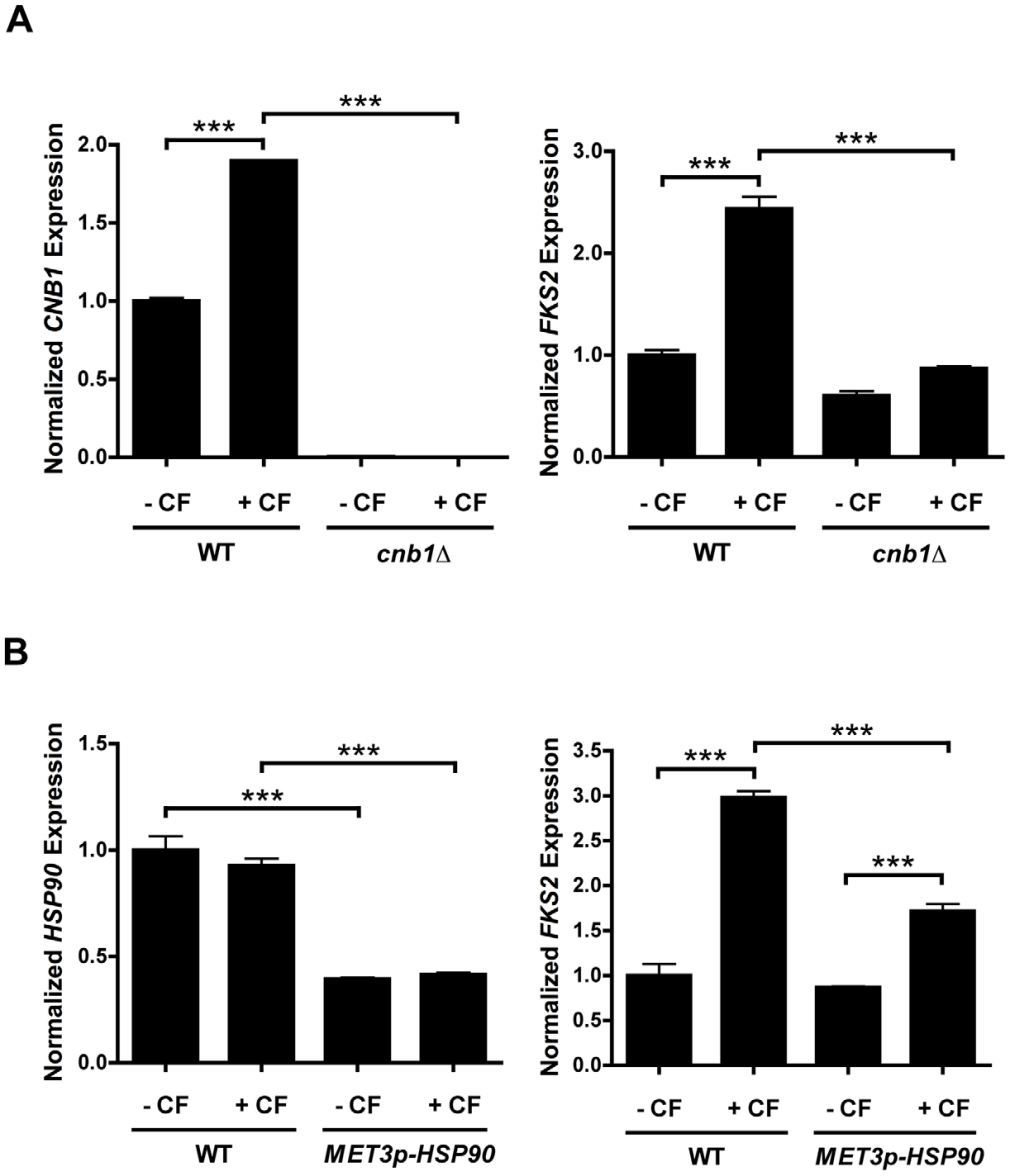
Caspofungin induces *FKS2* expression in a calcineurin- and Hsp90-dependent manner in *C. glabrata*. (**A**) Caspofungin induces expression of *CNB1*, which encodes the regulatory subunit of calcineurin, and *FKS2*, which encodes 1,3-β-D-glucan synthases, in the late clinical isolate G but not in its derivative lacking *CNB1*. Transcript levels of the regulatory subunit *CNB1* (left panel) and *FKS2* (right panel) were measured by quantitative RT-PCR after growth in rich medium at 30°C for one hour with or without caspofungin (CF) at 120 ng/ml, as indicated. Transcript levels are normalized relative to *ACT1*. Expression is relative to the untreated sample, which was set to 1. Data are means ± standard deviation for triplicate samples and representative of two independent biological replicate experiments. Three asterisks indicate *P*<0.001 (ANOVA, Bonferroni's Multiple Comparison Test). (**B**) Caspofungin induces expression of resistance determinant *FKS2* in the late clinical isolate G, but induction is reduced in its *MET3p-HSP90* derivative (*P*<0.001). Transcript levels of *HSP90* and *FKS2* were measured by quantitative RT-PCR after growth in conditions listed in (A), and in the presence of methionine at a concentration that had minimal impact on growth (0.25 µg/ml). Data are analyzed as in (A).

To test this model, we used quantitative RT-PCR to measure transcript levels of *FKS2*, encoding the catalytic subunit of 1,3-β-D-glucan synthase, in the echinocandin-resistant clinical isolate G and derivatives in which we deleted the regulatory subunit of calcineurin required for its function, encoded by *CNB1*, or in which we reduced *HSP90* levels. Transcript levels of *CNB1*, *HSP90*, and *FKS2* were monitored after growth in rich medium for one hour with or without caspofungin treatment. We found that caspofungin induced expression of both *CNB1* (*P*<0.001, ANOVA, Bonferroni's Multiple Comparison Test, Figure 8A) and *FKS2* (*P*<0.001, Figure 8A). Importantly, deletion of *CNB1* blocked caspofungin-induced upregulation of *FKS2* (*P*<0.001, Figure 8A). To reduce *HSP90* levels in the *MET3p-HSP90* strain, we included methionine in the medium for both this strain and the wild-type control at a concentration that had minimal impact on growth (0.25 µg/ml). *HSP90* levels were reduced in the *MET3p-HSP90* strain relative the wild-type control (*P*<0.001, Figure 8B). Caspofungin-dependent induction of *FKS2* was significantly reduced in the *MET3p-HSP90* strain (*P*<0.001, Figure 8B), suggesting that Hsp90 enables *FKS2* expression. Taken together, these results establish that *FKS2* is induced upon echinocandin exposure, and that full induction of the resistance determinant *FKS2* is dependent on both calcineurin and Hsp90. This provides a novel mechanism by which calcineurin and Hsp90 regulate echinocandin resistance in pathogenic fungi.

### *C. glabrata* petite mutants are not intrinsically resistant to echinocandins, but are refractory to the synergy between caspofungin and inhibitors of Hsp90 or calcineurin

**Figure 9:**
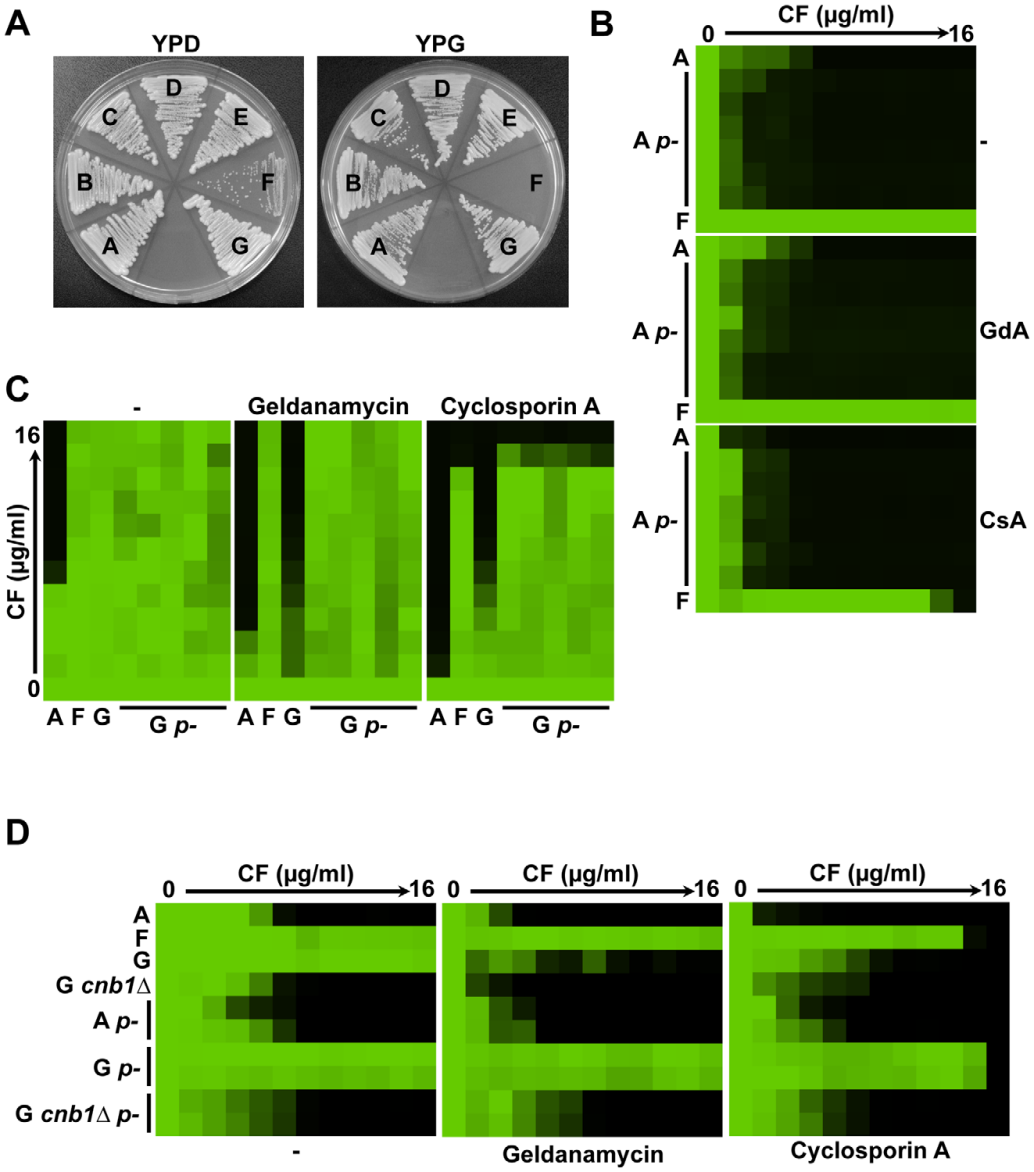
Clinical isolate F is a petite mutant and is refractory to the synergy between caspofungin and Hsp90 or calcineurin inhibitors. (**A**) Clinical isolate F is a petite mutant. Each clinical isolate from the *C. glabrata* echinocandin-resistant series was struck onto rich yeast extract-peptone-dextrose (YPD) agar medium or yeast extract-peptone-glycerol (YPG) agar medium which have glucose or glycerol as the sole carbon source, respectively. Isolate F produces small, transparent colonies on YPD and is unable to grow on YPG. Petite mutants have lost their mitochondrial function, are unable to respire, and therefore unable to utilize non-fermentable carbon sources. Isolates were patched onto the plates and photographed after incubation in the dark at 30°C for 24 hours. (**B**) The petite phenotype is not intrinsically involved in caspofungin (CF) resistance of *C. glabrata* clinical isolates. Petite mutants were generated from early clinical isolate A by growth in 10 µg/ml ethidium bromide for 48 hours in the dark and were identified based on morphology and inability to grow on YPG. CF resistance was then compared to resistant petite clinical isolate F in an MIC assay along with inhibition of Hsp90 with 20 µM geldanamycin (GdA) or inhibition of calcineurin with 10 µM cyclosporin A (CsA). (**C**) CF-resistant petite mutants of *C. glabrata* are refractory to the synergy between caspofungin and inhibitors of Hsp90 or calcineurin. Petite mutants were generated from late clinical isolate G as in part (B). CF resistance was then compared to the resistant petite clinical isolate F in an MIC assay with or without pharmacological inhibition of Hsp90 with 20 µM GdA or pharmacological inhibition of calcineurin with 10 µM CsA. (**D**) CF resistance of petite mutants of *C. glabrata* is dependent on calcineurin. Petite mutants were generated from the late clinical isolate G *cnb1Δ* deletion mutant as in part (B). Synergy between CF and Hsp90 inhibition with GdA (20 µM) or calcineurin inhibition with CsA (10 µM) was tested. MIC assays were performed and analyzed as in Figure 1.

Respiratory deficient mutants with loss of mitochondrial function, referred to as petite mutants, are associated with azole resistance in *S. cerevisiae*, *C. albicans*, and *C. glabrata* [98]–[102]. To date, there have been no reports of the petite phenotype contributing to echinocandin resistance, although *C. glabrata* is able to produce petite mutants at high frequency *in vitro* as well as *in vivo* [98], [101], [103]. Isolate F in our *C. glabrata* series was isolated from the patient at the same time point as isolate E, however, it was morphologically distinct and thus archived separately (Text S1). When cultured on rich medium containing dextrose as the carbon source, this isolate had a reduced growth rate relative to the other isolates and produced smaller more transparent colonies (Figure 9A), despite possessing the same karyotype (Figure S1) and complement of polymorphisms in the nuclear genome as isolate E (Figure 1). When cultured on rich medium containing glycerol as the carbon source, which is non-fermentable, isolate F was unable to grow (Figure 9A). These results suggests that isolate F is a petite mutant as it behaves morphologically as a petite and is unable to respire based on failure to grow on glycerol. Notably, isolate F had a distinct echinocandin resistance phenotype from the other resistant isolates in the series in that its resistance was maintained even in the presence of the Hsp90 inhibitors geldanamycin or radicicol (Figure 6), or the calcineurin inhibitor cyclosporin A (Figure 9).

To determine if the petite phenotype is intrinsically involved in echinocandin resistance of *C. glabrata* clinical isolates, petite mutants were generated from clinical isolate A and their resistance profiles were tested via MIC assays (Figure 9B). If the petite phenotype is sufficient to impart echinocandin resistance, then petite mutants generated from isolate A should acquire resistance, however, they do not (Figure 9B). This suggests that the petite phenotype is not intrinsically involved in *C. glabrata* echinocandin resistance.

To determine if petite mutants of *C. glabrata* are intrinsically refractory to the synergy between echinocandins and inhibitors of Hsp90 or calcineurin, petite mutants were generated from late clinical isolate G and their resistance profiles were tested via MIC assays (Figure 9C). The petite mutants generated from isolate G are indeed resistant to the combination of caspofungin and geldanamycin or cyclosporin A, suggesting that they either no longer require calcineurin or Hsp90 for *FKS2*-mediated resistance, or that they are simply resistant to geldanamycin and cyclosporin A. Notably, petite mutants are known to up-regulate multidrug efflux transporters [98], which may confer resistance to these pharmacological inhibitors by their increased efflux from the cell. To distinguish between these two possibilities, petite mutants were generated from the isolate G derivative in which calcineurin function was genetically compromised due to deletion of *CNB1*. If petite mutants no longer require calcineurin for echinocandin resistance, then deletion of *CNB1* should have no impact on resistance, however, this was not the case (Figure 9D). Petite mutants generated from isolate G *cnb1Δ* mutants were no longer resistant to caspofungin, suggesting that petite mutants are able to bypass the effects of cyclosporin A, and likely geldanamycin, potentially due to up-regulation of efflux pumps.

### Fungal burden of the *C. glabrata* clinical isolate series and *MET3p-HSP90* strain in a murine model of disseminated infection

**Figure 10:**
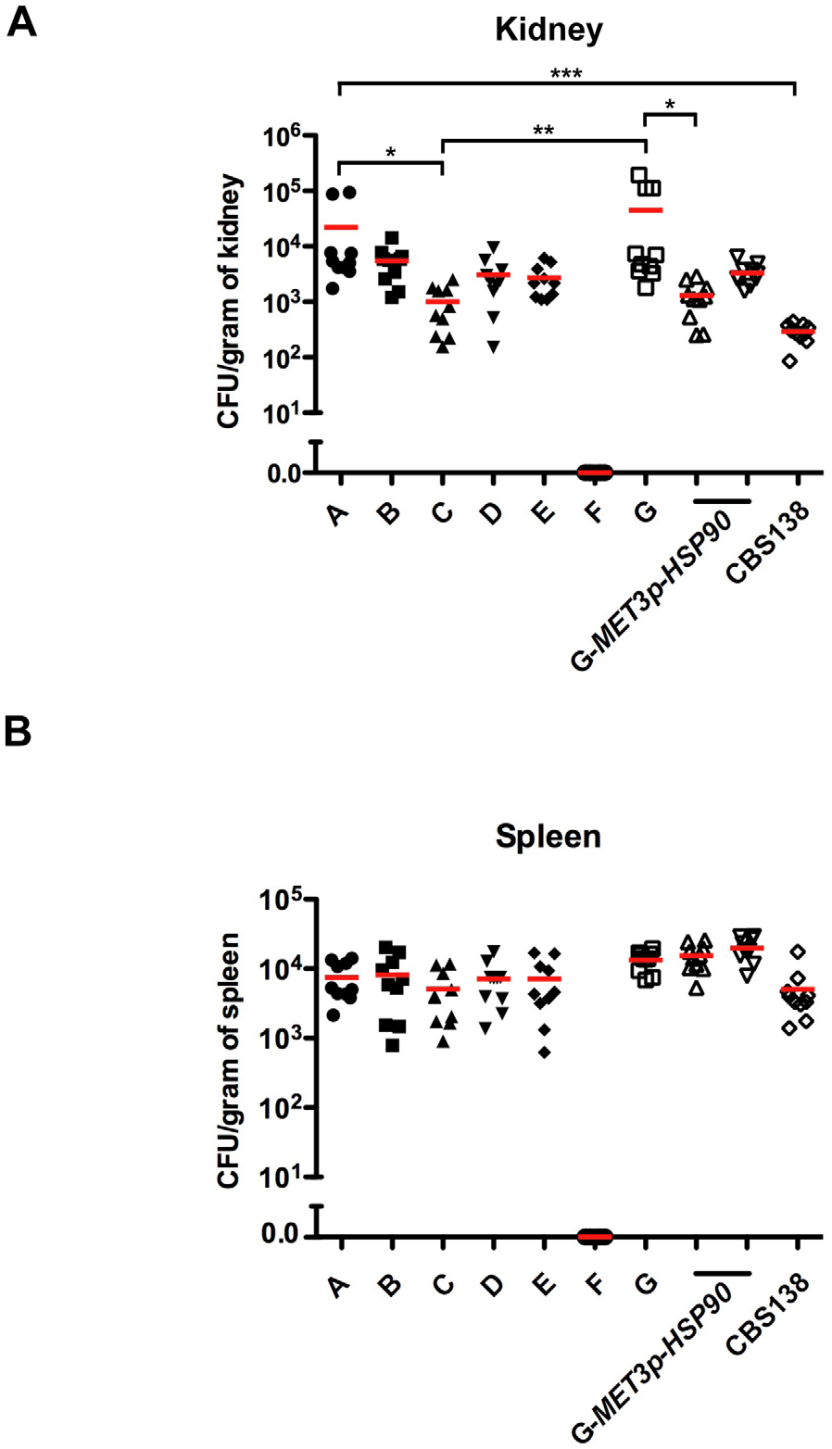
Fungal burden of the *C. glabrata* clinical isolate series and *MET3p-HSP90* strain in a murine model of disseminated infection. CD1 mice were infected with an inoculum of 2×10^7^ cells via lateral tail vein injection. One asterisk indicates *P*<0.05, two indicates *P*<0.01, and three indicates *P*<0.001 (Kruskal-Wallis Test, Dunn's Multiple Comparison). Fungal burden was measured in the (**A**) kidney and (**B**) spleen 7 days post infection. Organs of mice infected with isolate F were sterile.

We next turned to a murine model of disseminated candidemia to evaluate fitness of the series of clinical isolates and the impact of reduction of Hsp90 levels *in vivo*. Mice were infected by tail vein injection, and fungal burden in the kidney and spleen was assessed 7 days post infection. Infection with the initial clinical isolate A led to kidney fungal burden greater than that observed with the reference strain CBS138 (*P*<0.001, Kruskal-Wallis Test, Dunn's Multiple Comparison, Figure 10A). There was a trend towards reduced fitness, as measured by kidney fungal burden, associated with the early acquisition of echinocandin resistance (Figure 10A), consistent with the trend observed *in vitro* (Figure 2B). Aside from the petite mutant, isolate C showed the greatest reduction in fitness of all the clinical isolates in the series relative to isolate A (*P*<0.05, Figure 10A). This fitness cost was mitigated with further evolution as shown by an increase in kidney fungal burden of isolate G compared to isolate C (*P*<0.01, Figure 10A). Kidneys recovered from mice infected with the petite mutant (isolate F) were completely sterile (Figure 10A), consistent with the fitness deficit observed *in vitro* (Figure 2B) and findings of reduced virulence of some *C. glabrata* petite mutants [102], [104]. Methionine levels in the mouse are sufficient to repress gene expression from the *MET3* promoter in *C. albicans* leading to avirulence of conditional mutants [105], allowing us to test fungal burden of mice infected with the *MET3p-HSP90* strain. One of the two *MET3p-HSP90* derivatives of isolate G showed reduced kidney fungal burden relative to isolate G (*P*<0.05, Figure 10A), consistent with the importance of Hsp90 for growth *in vitro* and *in vivo* in a *C. albicans* murine model of systemic infection [106]. The second *MET3p-HSP90* derivative also showed a trend towards reduced kidney fungal burden relative to isolate G (Figure 10A). There were no significant differences observed among any of the isolates tested in the spleen with the exception of isolate F, for which the recovered spleens were sterile (Figure 10B), suggesting that most of the mutations that accumulated in the lineage have negligible impact on fitness in this environment, and that methionine levels are likely not sufficient to achieve substantial reduction of Hsp90 levels or that Hsp90 has a less important role for fungal proliferation in the spleen compared to the kidney. Thus, analysis of kidney fungal burden in a mouse model of disseminated infection reveals a trend towards reduced fitness accompanying early stages of echinocandin resistance that is ameliorated with further evolution, avirulence associated with a petite mutant, and the importance of Hsp90 for fitness in the host.

## Discussion

Our results provide the first global view of mutations that accompany the evolution of fungal drug resistance in a human host, implicate the premier compensatory mutation that ameliorates the fitness cost of echinocandin resistance, and suggest a new molecular mechanism regulating echinocandin resistance, with broad therapeutic potential. We report on *C. glabrata* bloodstream isolates that evolved increased resistance to the echinocandin caspofungin over a 10-month period during which the patient underwent multiple rounds of caspofungin treatment for recurrent candidemia (Figure S1 and Table S1). This case demonstrates that echinocandin resistance can evolve during treatment, and that an undetected nidus of infection may contribute to fungal persistence and drug resistance despite apparent adequate therapy and documentation of negative blood cultures after treatment, emphasizing the importance of routine antifungal susceptibility testing. Whole genome sequencing of the susceptible isolate recovered prior to drug treatment and the last resistant isolate revealed that 9 non-synonymous mutations accumulated during evolution in the human host (Table 1 and Figure 1). A mutation in *FKS2*, encoding the drug target, accompanied the largest increase in echinocandin resistance in the lineage; this mutation was sufficient to confer echinocandin resistance in a susceptible *C. glabrata* strain, but was associated with a fitness cost with reduced growth rate in the absence of drug (Figure 2). The fitness cost of resistance was ameliorated with further evolution, based on observations *in vitro* and in a murine model of systemic infection (Figures 2 and 10). The 8 additional mutations in genes not previously implicated in echinocandin resistance (Table 1) provide candidates for novel resistance determinants as well as mutations that mitigate the cost of resistance. Consistent with these possibilities, increased dosage of *CDC55*, which acquired a mutation accompanying the increase in fitness, mitigated the fitness cost of the *FKS2* mutation (Figure 3), while a mutation in *CDC6* that arose prior to the *FKS2* mutation was sufficient to confer a small increase in echinocandin resistance (Figure 5). Further, we establish that Hsp90 governs both basal tolerance to the echinocandins and *bona fide* resistance of clinical isolates (Figure 6), and that Hsp90 is important for *C. glabrata* proliferation in the mouse kidney (Figure 10). We found that calcineurin is a key mediator of Hsp90-dependent resistance (Figures 7). Hsp90 and calcineurin regulate echinocandin resistance by controlling expression of the resistance determinant *FKS2* (Figure 8), providing a novel mechanism via which Hsp90 and calcineurin govern echinocandin resistance in pathogenic fungi.

The whole genome sequence analysis yields powerful insights into the evolutionary dynamics of adaptation in the host, as well as novel mutations associated with resistance or with ameliorating the fitness cost of resistance. To date, mechanisms of echinocandin resistance remained restricted to mutations in the drug target. In the *C. glabrata* series, mutations in 4 genes not previously associated with echinocandin resistance (*MOH1*, *GPH1*, *CDC6*, and *TCB1/2*) accompanied an early and small increase in echinocandin resistance (Figure 1). Mutations in these genes could confer the small increase in resistance, or could create a genetic background in which the *FKS2* mutation is less detrimental; in the latter case, the mutation would have to confer a fitness benefit on its own in order to be selected for in advance of the *FKS2* mutation, or it could be selectively neutral and fixed by genetic drift. Given that a reduction in fitness accompanied the acquisition of these 4 mutations (Figure 2), it is likely that they contribute to resistance or were fixed by genetic drift. Consistent with the former possibility, polymorphisms in one of the genes that acquired a mutation associated with the small increase in resistance, *CDC6*, were common in an unrelated set of echinocandin-resistant *C. glabrata* isolates with Fks2 S663P (Figure 4), and the *CDC6-A511G(K171E)* mutation identified by whole genome sequencing was sufficient to confer a small increase in resistance (Figure 5). Mutations in 3 additional genes not previously implicated in echinocandin resistance (*DOT6*, *MRPL11*, and *SUI2*) coincided with the *FKS2* mutation, and a last mutation in *CDC55* arose in the last isolate, without any associated change in resistance (Figure 1). Given that the *FKS2* mutation is sufficient for the full resistance phenotype of the late clinical isolate (Figure 7), it is likely that these other mutations are unrelated to echinocandin resistance or that they mitigate the fitness cost of the *FKS2* mutation. It is notable that multiple mutations accumulated at the two major transitions in resistance (Figure 1). This is consistent with strong selection favouring the rapid accumulation of mutations in the lineage. That each of the mutations identified persisted throughout the lineage is consistent with the occurrence of selective sweeps, such that each mutation rose to near fixation. Selective sweeps in response to drug selection are also observed in experimental populations of *C. albicans* during the evolution of azole resistance *in vitro* [107].

The fate of drug-resistant mutants in nature depends on their fitness relative to drug-susceptible counterparts. While resistance mutations are expected to confer a fitness benefit in the presence of the drug, they may also confer a cost in terms of reduced fitness in the absence of the drug. This model is consistent with the impact of the *FKS2* T1987C mutation observed in the *C. glabrata* lineage, and reported for target-mediated echinocandin resistance in *C. albicans* [88]. This mutation confers a major increase in growth in the presence of echinocandin (Figure 2A), but also confers reduced growth in the absence of the drug (Figure 2B). The deleterious impact on fitness is likely due to the reduced catalytic capacity commonly observed among 1,3-β-D-glucan synthase enzymes that acquire amino acid substitutions that reduce their sensitivity to echinocandins [36]. *C. glabrata* may upregulate *FKS2* expression to compensate for its decreased catalytic capacity [36], or may acquire additional mutations that mitigate the cost of the resistance mutation. The fitness effects of antibiotic resistance mutations have been studied extensively in bacteria, where most resistance mechanisms are associated with a fitness cost that manifests in reduced growth rate [84]. In the vast majority of cases, the fitness cost is mitigated by the acquisition of compensatory mutations [84]–[87]. Consistent with these patterns, any cost of resistance in experimental populations of *C. albicans* that evolved azole resistance *in vitro* was mitigated was further evolution [108], as many changes in gene expression observed in the less fit, resistant population were restored to the ancestral state [15]. In the *C. glabrata* lineage studied here, a mutation in *CDC55* accompanied an increase in fitness of isolate G relative to isolate D (Figures 1 and 2). Increased dosage of *CDC55* ameliorated fitness of an independent *FKS2* T1987C mutant, independent of whether it was the *C463T(P155S)* allele (Figure 3). This suggests that the *C463T(P155S)* mutation may confer increased Cdc55 function and that fitness can be ameliorated by either elevated activity or dosage of Cdc55. This *C463T(P155S)* mutation was not identified in other *C. glabrata* echinocandin-resistant *FKS2* T1987C mutants (Figure 4), suggesting that distinct compensatory mutations might be favoured *in vivo* or that they may harbor duplications of *CDC55*. The mechanisms by which alterations in *CDC55* ameliorates fitness of the *FKS2* mutant and the scope of beneficial effects in other backgrounds remains to be determined.

Morphological variants that emerge in an evolutionary lineage can reveal important features of mechanisms of drug resistance or drug synergy. Isolate F is a petite mutant based on morphology and inability to grow on a non-fermentable carbon source (Figure 9A). Such respiratory deficient mutants with loss of mitochondrial function are associated with azole resistance in *S. cerevisiae*, *C. albicans*, and *C. glabrata* [98]–[102]. The azole resistance of petites is attributed to increased expression of multidrug transporters of the ATP binding cassette family [98], [99], [101]. The petite phenotype has not been linked to echinocandin resistance to date, consistent with the limited evidence that multidrug transporters are involved in resistance to this drug class [109]–[112]. Indeed, induction of petite mutants in the early *C. glabrata* clinical isolate A does not confer echinocandin resistance, confirming that petite mutants are not intrinsically resistant to echinocandins (Figure 9B). In *S. cerevisiae*, several mitochondrial proteins have been identified as required for echinocandin tolerance [113], consistent with the slight reduction in tolerance we observe in *C. glabrata* petite mutant derivatives of isolate A (Figure 9). A striking feature of the isolate F is that its echinocandin resistance phenotype is recalcitrant to the impact of the Hsp90 inhibitor geldanamycin or calcineurin inhibitor cyclosporin A, unlike that of all other isolates in the series (Figure 9C). Induction of petite mutants in late clinical isolate G confirms that the petite phenotype is intrinsically recalcitrant to the impact of geldanamycin or cyclosporin A (Figure 9C). Genetic compromise of calcineurin function in petite mutants abrogates echinocandin resistance, suggesting that petites are simply resistant to cyclosporin A (Figure 9D), and likely geldanamycin; this may be attributable to overexpression of multidrug transporters in petites that remove these inhibitors from the cell. Whether the original isolate F petite mutant arose during evolution in the human host or during sampling remains unknown, although our finding that kidneys and spleens from mice infected with the petite mutant were completely sterile (Figure 10), suggests that petite mutant arose shortly before or during its isolation. In contrast, one *C. glabrata* petite mutant was reported to have enhanced virulence relative to an isolate recovered from the same patient at an earlier time point, however, an isogenic control was lacking [102]. Consistent with our findings, most *C. glabrata* petite mutants have been reported to have attenuated virulence relative to isogenic controls [104].

Our findings of reduced kidney fungal burden in mice infected with the late clinical isolate G with conditional expression of *HSP90* driven by the *MET3* promoter support a role for Hsp90 in proliferation in the host (Figure 10). That there was only a modest reduction in fungal burden is surprising in light of Hsp90's essentiality *in vitro*, as observed by methionine-mediated transcriptional repression (Figure 6 and 8). It is likely that methionine levels in the mouse were not sufficient to repress the *C. glabrata MET3* promoter and deplete Hsp90, and that methionine levels were even lower in the spleen, leading to no significant differences in fungal burden (Figure 10). Notably, methionine levels in the mouse were sufficient to cause avirulence of an *ino1Δ/ino1Δ itr1*Δ/*MET3p-ITR1* conditional mutant of *C. albicans* [105], but only partial attenuation of virulence of a *C. albicans* conditional mutant of the essential gene *FBR1* (*fbr1*Δ/*MET3p-FBR1*) [114]. The impact on virulence using the *MET3* system to deplete essential genes may depend on the level of depletion required to observe phenotypic effects. Using a tetracycline-repressible promoter system and delivery of tetracycline to the mice in a systemic model of infection, Hsp90 has been shown to be required for proliferation of *C. albicans* [106], suggesting that this promoter system may be more suitable for *in vivo* studies given that doses of tetracycline can be titrated.

Our results further establish Hsp90 and calcineurin as the first regulators of *bona fide* echinocandin resistance in *C. glabrata*, and reveal that resistance circuitry has been rewired over evolutionary time. The molecular chaperone Hsp90 and its client protein calcineurin govern basal tolerance and resistance to both the azoles and the echinocandins in *C. albicans* [41], [44], [47]. Pharmacological inhibition of Hsp90 can enhance the efficacy of azoles against *C. glabrata* [115], and calcineurin plays an important role in both azole and echinocandin tolerance [49]. The roles of Hsp90 and calcineurin in azole resistance are conserved in *S. cerevisiae* [44], [47]. However, compromise of Hsp90 or calcineurin function does not alter echinocandin susceptibility in *S. cerevisiae* under any of the standard conditions tested where echinocandin susceptibility of *C. albicans* is affected [41]. Here, we find that Hsp90 and calcineurin are required for basal tolerance to echinocandins in *C. glabrata* as well as for resistance that evolves in a human host (Figures 4 and 5), suggesting that despite the closer evolutionary relationship of *C. glabrata* to *S. cerevisiae*, the *C. glabrata* cellular circuitry governing resistance to drugs that target the cell wall shares more similarity to that of its more distant pathogenic relative, *C. albicans*. While conditions may exist in which Hsp90 and calcineurin influence echinocandin susceptibility in *S. cerevisiae*, it is clear that the *C. glabrata* phenotypic response more closely resembles that of *C. albicans* than *S. cerevisiae*. Notably, signaling pathways governing cell wall integrity have been rewired between *C. albicans* and *S. cerevisiae* [116]. The cell wall is essential for fungal viability and is an elaborate structure, components of which are recognized by vigilant immune cells in the human host [117]. As commensals and opportunistic pathogens, *C. glabrata* and *C. albicans* are likely to harbour circuitry governing cell wall architecture that is subject to strong selection in response to host immune system challenge.

This work establishes that targeting Hsp90 or calcineurin has broad therapeutic potential for infections caused by one of the leading fungal pathogens of humans, and complements the expanding repertoire of therapeutic applications for inhibitors of Hsp90 and calcineurin in the treatment of infectious disease. Inhibition of Hsp90 or calcineurin transforms echinocandins from ineffective to highly efficacious against echinocandin-resistant *C. glabrata* (Figures 6 and 7). Notably, a human recombinant antibody against Hsp90 also has synergistic activity with echinocandins against *C. glabrata* in a mouse model [118], though the mechanism by which this antibody works remains entirely unknown as it is unlikely to enter intact fungal cells to influence function of the cytosolic Hsp90 chaperone. Genetic compromise of Hsp90 function enhances the efficacy of azoles and echinocandins in a mouse model of systemic *C. albicans* infection [41], [45]. Genetic or pharmacological compromise of Hsp90 also transforms fluconazole from ineffective to highly efficacious against *C. albicans* biofilms in a mammalian model of biofilm infection [119]. Beyond *Candida* species, inhibition of Hsp90 also enhances antifungal efficacy against the most lethal mould, *A. fumigatus*, in biofilms and in a metazoan model of infection [45], [119]. Consistent with the functional relationship between Hsp90 and calcineurin, calcineurin inhibitors also have therapeutic potential and are synergistic with azoles against *C. albicans* endocarditis, keratitis, and biofilms in mammalian models [120]–[122]. Beyond their utility in the treatment of fungal infections, Hsp90 and calcineurin are promising targets for treating infections caused by protozoan parasites including *Plasmodium falciparum*, *Trypanosoma evansi*, and *Leishmania major* [123]–[126]. Supporting their clinical relevance, Hsp90 inhibitors have advanced in clinical trials for the treatment of cancer and other diseases [9], [127], [128], and calcineurin inhibitors are widely used in the clinic as immunosuppressants [92]. Given the potential for toxicity upon inhibition of key cellular regulators in the host during infection [45], the challenge for further development of Hsp90 and calcineurin as therapeutic targets for infectious disease lies in developing pathogen-selective inhibitors or drugs that target pathogen-specific components of the cellular circuitry governing drug resistance and virulence.

## Materials and Methods

### Ethics statement

Dr. Susan M. Poutanen discussed this study and specifically highlighted the inclusion of the case history and the case isolates in this study with Nushrat Sultana, Research Ethics Board Coordinator for the Research Ethics Board at Mount Sinai Hospital in Toronto, Canada, the hospital in which the case patient had been hospitalized. Nushrat Sultana confirmed that completing and publishing a case study that involves only a single case does not require review by the Research Ethics Board. Written confirmation has been provided by Dr. Ronald Heslegrave, Chair, Research Ethics Board, Mount Sinai Hospital. Oral and written consent was provided by the case patient's mother, as the patient is deceased. Animals studies conducted in the Division of Laboratory Animal Resources (DLAR) facilities at Duke University Medical Center (DUMC) were handled with good practice as defined by the United States Animal Welfare Act and in full compliance with the guidelines of the DUMC Institutional Animal Care and Use Committee (IACUC). The murine systemic infection model was reviewed and approved by the DUMC IACUC under protocol number A238-09-08.

### Patient history for *C. glabrata* clinical isolate series

A 43 year-old female had four episodes of *Candida glabrata* candidemia between April 2004 and October 2005. Her past medical history was significant for severe fistulizing Crohn's disease diagnosed at the age of 9 years. She had been on total parenteral nutrition (TPN) since 2003 for short gut syndrome due to numerous small bowel resections over the 30-year course of her disease.

#### April 2004

The patient was admitted to hospital with generalized malaise, fever, and *Clostridium difficile* infection associated diarrhea (CDAD). Admission blood cultures demonstrated *C. glabrata* candidemia with evidence of pulmonary nodules on thorax computerized tomography (CT). No evidence of endophthalmitis was noted on ophthalmologic exam. Her two central venous catheters were removed and caspofungin was started at 70 mg od×1 then 50 mg od intravenously with clinical response. Cultures of the catheter tip and a repeat set of blood cultures were negative. After 3 weeks of treatment, she was discharged with plans to continue caspofungin until resolution of the pulmonary nodule on repeat thorax CT. An outpatient echocardiogram was also ordered. The patient was non-adherent with outpatient follow-up but had three sets of negative blood cultures drawn while off caspofungin therapy during emergency department visits 6 months after discharge.

#### January 2005

The patient presented to the hospital with a 1-week history of fatigue, nausea, vomiting and diarrhea, and was diagnosed with TPN-associated cholestasis. Three out of four sets of blood cultures drawn at admission grew *C. glabrata*. Eye exams revealed no evidence of endophthalmitis. Her central venous catheter was removed and she was restarted on caspofungin at the same dose. Four sets of repeat blood cultures were negative. She continued to have intermittent fevers and one week after admission was transferred to the intensive care unit with septic shock and intubated for hypoxic respiratory distress. A thorax CT demonstrated bilateral airspace consolidation consistent with acute respiratory distress syndrome and an abdominal CT demonstrated a fistulous tract to a 5 cm×0.3 cm anterior abdominal wall collection. The patient was treated with piperacillin-tazobactam with an excellent clinical response. Piperacillin-tazobactam was ordered to continue until the collection resolved. Caspofungin was stopped two weeks after the last positive culture for *C. glabrata*.

#### February 2005

One week after the caspofungin was stopped while continuing on piperacillin-tazobactam, the patient presented with recrudescence of fever and abdominal pain. Five out of five sets of blood cultures grew *C. glabrata*. Eye exam and transthoracic echocardiogram revealed no evidence of endophthalmitis or endocarditis. Her central venous catheter was removed and the catheter tip was positive for *Candida* species, not *C. albicans*. Caspofungin was restarted at the same dose with good clinical response. Two sets of repeat blood cultures were negative. Caspofungin was continued for a total of 4 weeks and the patient was discharged. The patient was not adherent to outpatient follow-up appointments but multiple repeat blood cultures drawn at the TPN clinic over the following six months off caspofungin were negative.

#### October 2005

The patient presented to the emergency department with a lower gastrointestinal bleed and shock and was immediately transferred to the intensive care unit. She was empirically started on piperacillin-tazobactam and fluconazole for presumed intra-abdominal sepsis but died 3 days after admission. Three sets of ante-mortem blood cultures grew *C. glabrata*.

### Strains and culture conditions

Archives of *C. glabrata* strains were maintained at −80°C in 25% glycerol. Strains were grown in either YPD (1% yeast extract, 2% bactopeptone, 2% gucose), YPG (1% yeast extract, 2% bactopeptone, 2% glycerol), synthetic defined medium (0.67% yeast nitrogen base, 2% glucose) supplemented with required amino acids, or RPMI medium 1640 (Gibco, 3.5% MOPS, 2% glucose, pH 7.0). 2% agar was added for solid media. Strains used in this study are listed in Table S2. Strain construction is described in Text S1.

### Plasmid construction

Recombinant DNA procedures were performed according to standard protocols. Plasmids used in this study are listed in Table S3. Plasmid construction is described in the Text S1. Plasmids were sequenced to verify the absence of any nonsynonymous mutations. Primers used in this study are listed in Table S4.

### Pulsed-field Gel Electrophoresis (PFGE) karyotyping and restriction enzyme-PFGE

PFGE-karyotyping and restriction enzyme-PFGE using SfiI were performed following previously reported methods [129].

### Whole genome sequencing

Genomic DNA was extracted from clinical isolate A and G, and sequencing libraries were prepared using the Illumina genomic DNA library preparation kit according to the manufacturers recommendations (Illumina, CA) with several modifications. In brief, DNA was sheared by sonication to an average fragment length of 200 base pairs. Illumina adapters were blunt-end ligated and libraries were amplified by PCR and purified using Ampure (Agencourt) beads at a DNA:bead ratio of 1:0.9. Each sample was sequenced together in a single lane on an Illumina Genome Analyzer II platform, yielding 5.1 and 3.8 million 76 base pair single-end reads for isolate A and isolate G, respectively, resulting in 22 to 30× genome coverage. Reads were aligned using SOAP2 (PMID: 19497933) against the reference genome sequence of CBS138 [57]. Single nucleotide variants were identified using a machine learning approach as described previously [130]. All non-synonymous mutations identified were validated independently using Sanger sequencing. Raw data is available for download from the Short Read Archive under accession SRA047280.2.

### Mutation mapping

The 9 non-synonymous mutations identified by whole genome sequencing were mapped across clinical isolates B, C, D, E, and F using Sanger sequencing. *CgFKS2* was amplified using oLC1344/1345, *CgDOT6* with oLC1559/1560, *CgMOH1* with oLC1561/1562, *CgGPH1* with oLC1563/1564, *CgMRPL11* with oLC1565/1566, *CgCDC6* with oLC1567/1568, *CgCDC55* with oLC1569/1570, *CgSUI2* with oLC1571/1572, and *CgTCB1/2* with oLC1573/1574. *CgFKS2* was sequenced with oLC1344, *CgDOT6* with oLC1559, *CgMOH1* with oLC1561, *CgGPH1* with oLC1563, *CgMRPL11* with oLC1565, *CgCDC6* with oLC1567, *CgCDC55* with oLC1569, *CgSUI2* with oLC1571, and *CgTCB1/2* with oLC1573.

### Minimum inhibitory concentration assays

Antifungal susceptibility was determined in flat bottom, 96-well microtiter plates (Sarstedt) using a modified broth microdilution protocol, as described [44]. Minimum inhibitor concentration (MIC) tests were set up in a total volume of 0.2 ml/well with 2-fold dilutions of caspofungin (CF, generously provided by Rochelle Bagatell). Echinocandin gradients were from 16 µg/ml down to 0 with the following concentration steps in µg/ml: 16, 8, 4, 2, 1, 0.5, 0.25, 0.125, 0.0625, 0.03125, 0.015625, and 0. Cell densities of overnight cultures were determined and dilutions were prepared such that ∼10^3^ cells were inoculated into each well. Geldanamycin (GdA, A.G. Scientific, Inc.) and radicicol (RAD, A.G. Scientific, Inc.) were used to inhibit Hsp90 at the indicated concentrations, and cyclosporin A (CsA, CalBiochem) and FK506 (A.G. Scientific, Inc.) were used to inhibit calcineurin at the indicated concentrations. Dimethyl sulfoxide (DMSO, Sigma Aldrich Co.) was the vehicle for GdA, RAD, CsA, and FK506. Sterile water was the vehicle for CF. Plates were incubated in the dark at 30°C for the time period indicated, at which point plates were sealed and re-suspended by agitation. Absorbance was determined at 600 nm using a spectrophotometer (Molecular Devices) and was corrected for background from the corresponding medium. Each strain was tested in duplicate on at least two occasions. MIC data was quantitatively displayed with colour using the program Java TreeView 1.1.3 (http://jtreeview.sourceforge.net).

Clinical antifungal MICs were determined using broth microdilution with RPMI 1640 broth for amphotericin, fluconazole, ketoconazole, itraconazole, voriconazole, and caspofungin following Clinical and Laboratory Standards Institute document M27-A3 [56]. Visual MIC endpoints were read after 24 hours of incubation at 35°C for caspofungin and after 48 hours of incubation for all other drugs. Complete inhibition was used to determine amphotericin endpoints; 50% inhibition (compared to growth control) was used for caspofungin and 80% inhibition was used for the other drugs.

### Quantitative reverse transcription PCR (qRT-PCR)

To measure gene expression changes in response to caspofungin treatment in *C. glabrata*, cells were grown overnight in YPD at 30°C. Cells were diluted to OD_600_ of 0.2 in SD and grown for 2 hours in duplicate for each strain at 25°C. After 2 hours of growth 120 ng/ml CF was added to one of the two duplicate cultures and left to grow for one additional hour at 25°C. Cells were centrifuged and pellets were frozen at −80°C immediately. RNA was isolated using the QIAGEN RNeasy kit and RNAse-free DNase (QIAGEN), and cDNA synthesis was performed using the AffinityScript cDNA synthesis kit (Stratagene). PCR was performed using SYBR Green JumpStart Taq ReadyMix (Sigma-Aldrich Co) with the following cycling conditions: 94°C for 2 minutes, 94°C for 15 seconds, 60°C for 1 minute, 72°C for 1 minute, for 40 cycles. All reactions were performed in triplicate, using primers for the following genes: *CgACT1* (oLC1500/1501), *CgCNB1* (oLC1502/1503), and *CgFKS2* (oLC1498/1499). Data were analyzed using iQ5 Optical System Software Version 2.0 (Bio-Rad Laboratories, Inc). Statistical significance was evaluated using GraphPad Prism 5.0.

To monitor gene expression changes in response to reduction of *HSP90* levels, strains CgLC751 and CgLC2121 were grown overnight at 30°C in synthetic defined medium supplemented with 0.25 µg/ml methionine. Stationary phase cultures were diluted to an OD600 of 0.2 and grown for 2 hours at 30°C in synthetic defined medium with methionine. Following incubation, cultures were split and 120 ng/ml caspofungin added to one set. Cells were harvested after one hour and RNA was isolated using the QIAGEN RNeasy kit and cDNA synthesis was performed using the AffinityScript cDNA synthesis kit (Stratagene). PCR was carried out using the SYBR Green Fast Mix (Applied Biosystems) with the following cycle conditions: 95°C for 20 seconds, and 95°C for 3 seconds, 60°C for 30 seconds, for 40 cycles. All reactions were done in triplicate using the following primer pairs: *CgACT1* (oLC1500/1501), *CgHSP90* (oLC2155/2156), *CgFKS2* (oLC1498/1499). Data were analyzed in the StepOne analysis software (Applied Biosystems).

### Growth curves

Growth kinetics were measured in *C. glabrata* strains by inoculating cells from an overnight culture grown in YPD at 30°C to an OD_600_ of 0.0625 in 100 µl of RPMI with 2% glucose in flat bottom, 96-well microtiter plates (Sarstedt). Cells were grown in a Tecan GENios microplate reader (Tecan Systems Inc., San Jose, USA) at 37°C with orbital shaking. Optical density measurements (OD_600_) were taken every 15 minutes for 48 hours. Statistical significance was evaluated using GraphPad Prism 4.0. For assays involving plasmids, selection was maintained with 150 µg/ml nourseothricin (Werner BioAgents).

### Generation of petite mutants of *C. glabrata*

*C. glabrata* strains were inoculated from solid YPD medium to liquid YPD medium containing 10 µg/ml ethidium bromide (EtBr). The culture was grown overnight, shaking at 30°C in the dark. Approximately 100 cells were plated on YPD agar to isolate single colonies. After 2 days of incubation at 30°C, single colonies were tested for growth on YPD agar and YP-glycerol agar. Colonies able to grow on glucose as the sole carbon source but not on glycerol as the sole carbon source were selected.

### Murine model of systemic infection

Four- to five-week-old male CD1 mice from The Jackson Laboratory (n = 10 for each group) were utilized in this study. *C. glabrata* strains were grown in 10 ml liquid methionine-free minimal medium (6.7 g yeast nitrogen base without amino acids and 20 g glucose in 1 liter) overnight at 30°C. Cultures were washed twice with 10 ml of phosphate buffered saline (PBS), and the cells were then resuspended in 2 ml of PBS. Cells were counted with a hemocytometer and resuspended in an appropriate amount of PBS to obtain an infection inocula concentration of 1×10^8^ cells/ml. Two hundred microliters (2×10^7^ cells) were used to infect mice by lateral tail vein injection. Appropriate dilutions of the cells were plated onto solid methionine-free minimal medium and incubated at room temperature for 48–96 hours to confirm cell viability. *C. glabrata* infected mice were sacrificed and dissected on day 7 post-infection. The kidney and spleen tissues were removed, weighed, transferred to a 15 ml Falcon tube filled with 5 ml PBS, and homogenized for 10 seconds at 17,500 rpm (Power Gen 500, Fisher Scientific). Tissue homogenates were serially diluted, and 100 µl was plated onto YPD solid medium (except CgLC2121 and CgLC2122 strains which were plated onto methionine-free minimal medium containing 100 µg/ml chloramphenicol). The plates were incubated at room temperature for 48–96 hours to determine CFUs per gram of organ. We confirmed that organ-recovered CgLC2121 and CgLC2122 cells only grew on methionine-free minimal medium but not on medium containing methionine. All experimental procedures were carried out according to NIH guidelines and Duke IACUC protocols for the ethical treatment of animals.

### Accession numbers (NCBI Entrez Gene ID number)

*C. glabrata: ACT1* (2890423); *CDC55* (2890752); *CDC6* (2890231); *CNB1* (2890566); *DOT6* (2886442); *FKS1* (2888318); *FKS2* (2890040); *FKS3* (2891236); *GPH1* (2887861); *HSP90* (2891108); *MET3* (2886500); *MOH1* (2887590); *MRPL11* (2889720); *RHO1* (2888920); *SUI2* (2886561); *TCB1/2* (2889714).

*S. cerevisiae*: *CDC55* (852685); *DOT6* (856822); *FKS1* (851055); *FKS2* (852920); *FKS3* (855353); *GPH1* (856289); *MOH1* (852231); *MRPL11* (851325); *RHO1* (856294); *SUI2* (853463); *TCB1* (854253); *TCB2* (855637).

## Supporting Information

Figure S1 ***C. glabrata*** **clinical isolates serially isolated from a patient are of the same lineage.** Pulsed-field gel electrophoresis (PFGE) karyotype analysis reveals that isolates A through G, inclusive, are related and likely of the same lineage. All samples were run on the same gel and the picture was cropped to order the isolates. Lanes ‘M’ contain a marker, lanes 1 and 2 are of two control *C. glabrata* strains and lanes A through G are of isolate A through isolate G, in the same order.
(TIF)

Figure S2 **Hsp90 and calcineurin play critical roles in echinocandin resistance of** ***C. glabrata*** **in RPMI medium.** Pharmacological inhibition of Hsp90 with geldanamycin (GdA) or pharmacological inhibition of calcineurin with cyclosporin A (CsA) reduces caspofungin (CF) resistance of *C. glabrata* clinical isolates in an MIC assay. Isolates are arranged in the same order as they were recovered from a patient who was on CF treatment, where isolate A was recovered pre-treatment and isolate G was recovered after multiple rounds of CF treatment. Assays were done in RPMI medium supplemented with 2% glucose at 30°C for 72 hours. Optical densities were averaged for duplicate measurements and normalized relative to CF-free controls.
(TIF)

Table S1 ***In Vitro*** **susceptibility of** ***C. glabrata*** **clinical isolates to clinically relevant antifungal drugs.** Antifungal minimum inhibitory concentrations (MICs) for serially isolated *C. glabrata* clinical isolates were determined using broth microdilution with RPMI 1640 broth for amphotericin, fluconazole, ketoconazole, itraconazole, voriconazole, and caspofungin following Clinical and Laboratory Standards Institute document M27-A3. Isolates are arranged in the same order as they were recovered from the patient, where isolate A was recovered pre-treatment and isolate G was recovered after multiple rounds of caspofungin treatment. MIC endpoints (µg/ml) were read after 24 hours of incubation at 35°C for caspofungin and after 48 hours of incubation for all other drugs. Complete inhibition was used to determine amphotericin endpoints; 50% inhibition (compared to a drug-free growth control) was used for caspofungin and 80% inhibition was used for other drugs.
(DOC)

Table S2 ***Candida glabrata*** **strains used in this study.**
(DOC)

Table S3 **Plasmids used in this study.**
(DOC)

Table S4 **Primers used in this study.**
(DOC)

Text S1 **Supporting** **Materials and Methods** **.**
(DOC)
