# Supplementary figures and images for: Global Analysis of the Evolution and Mechanism of Echinocandin Resistance in Candida glabrata
Source: PLoS Pathog. 2012 May 17;8(5):e1002718. doi: 10.1371/journal.ppat.1002718 (PMC3355103; doi:10.1371/journal.ppat.1002718)

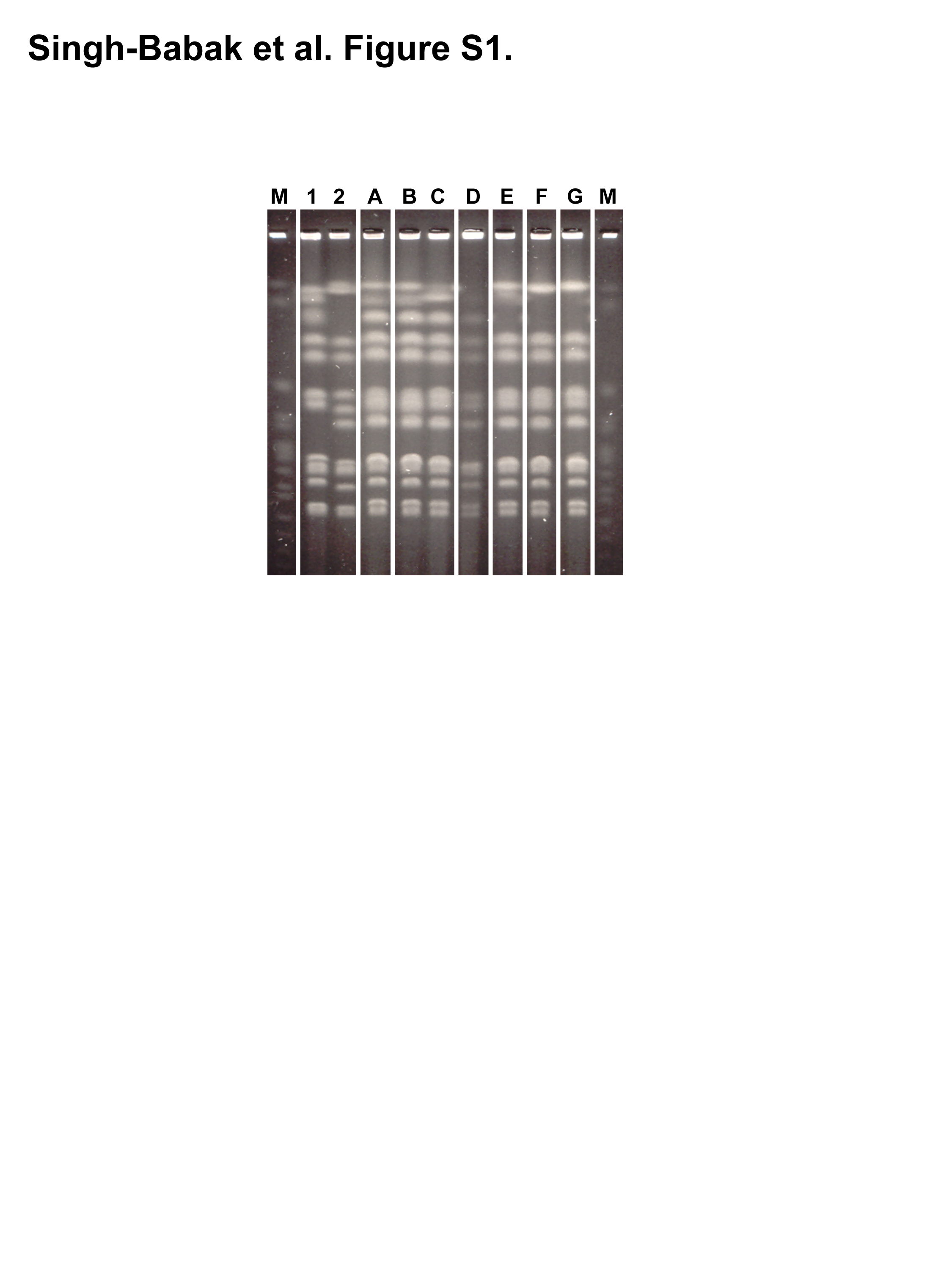

Supplement: Figure S1 — C. glabrata clinical isolates serially isolated from a patient are of the same lineage. Pulsed-field gel electrophoresis (PFGE) karyotype analysis reveals that isolates A through G, inclusive, are related and likely of the same lineage. All samples were run on the same gel and the picture was cropped to order the isolates. Lanes ‘M’ contain a marker, lanes 1 and 2 are of two control C. glabrata strains and lanes A through G are of isolate A through isolate G, in the same order. (TIF) [file ppat.1002718.s001.tif]

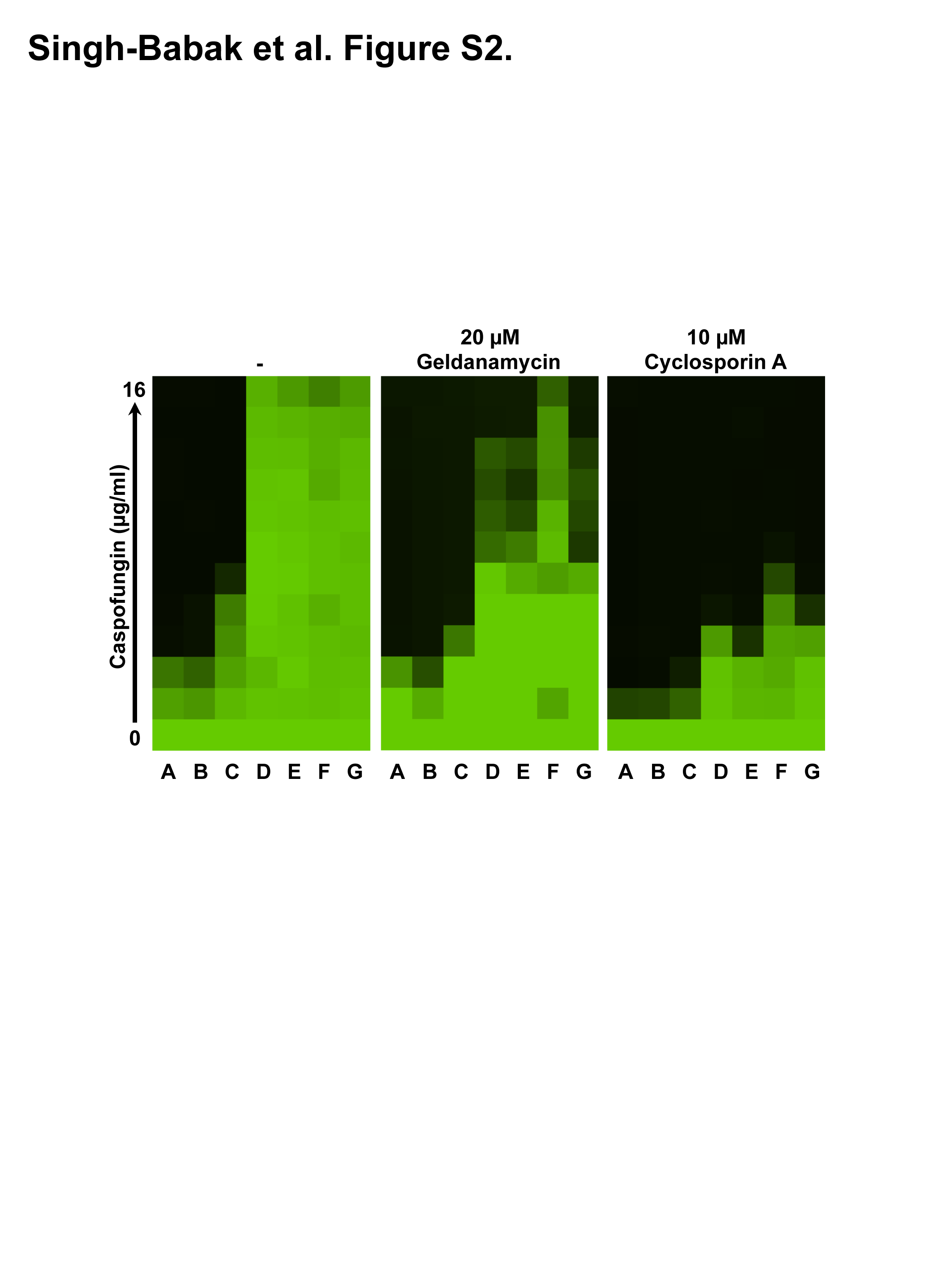

Supplement: Figure S2 — Hsp90 and calcineurin play critical roles in echinocandin resistance of C. glabrata in RPMI medium. Pharmacological inhibition of Hsp90 with geldanamycin (GdA) or pharmacological inhibition of calcineurin with cyclosporin A (CsA) reduces caspofungin (CF) resistance of C. glabrata clinical isolates in an MIC assay. Isolates are arranged in the same order as they were recovered from a patient who was on CF treatment, where isolate A was recovered pre-treatment and isolate G was recovered after multiple rounds of CF treatment. Assays were done in RPMI medium supplemented with 2% glucose at 30°C for 72 hours. Optical densities were averaged for duplicate measurements and normalized relative to CF-free controls. (TIF) [file ppat.1002718.s002.tif]
