# Supplementary material for: Global Analysis of the Evolution and Mechanism of Echinocandin Resistance in Candida glabrata
Source: PLoS Pathog. 2012 May 17;8(5):e1002718. doi: 10.1371/journal.ppat.1002718 (PMC3355103; doi:10.1371/journal.ppat.1002718)
Supplement: Table S1 — In Vitro susceptibility of C. glabrata clinical isolates to clinically relevant antifungal drugs. Antifungal minimum inhibitory concentrations (MICs) for serially isolated C. glabrata clinical isolates were determined using broth microdilution with RPMI 1640 broth for amphotericin, fluconazole, ketoconazole, itraconazole, voriconazole, and caspofungin following Clinical and Laboratory Standards Institute document M27-A3. Isolates are arranged in the same order as they were recovered from the patient, where isolate A was recovered pre-treatment and isolate G was recovered after multiple rounds of caspofungin treatment. MIC endpoints (µg/ml) were read after 24 hours of incubation at 35°C for caspofungin and after 48 hours of incubation for all other drugs. Complete inhibition was used to determine amphotericin endpoints; 50% inhibition (compared to a drug-free growth control) was used for caspofungin and 80% inhibition was used for other drugs. (DOC) [file ppat.1002718.s003.doc]

**Table S1. *In vitro* susceptibility of *C. glabrata* clinical isolates to clinically relevant antifungal drugs (µg/ml).**

|  | **A** | **B** | **C** | **D** | **E** | **F** | **G** |
| --- | --- | --- | --- | --- | --- | --- | --- |
| **Date of Isolation** | April 7, 2004 | April 14, 2004 | January 12, 2005 | January 13, 2005 | February 7, 2005 | February 7, 2005 | February 11, 2005 |
| **Amphotericin** | 0.5 | 1 | 0.5 | 1 | 0.5 | 1 | 0.5 |
| **Fluconazole** | 2 | 4 | 4 | 16 | 8 | >64 | 8 |
| **Ketoconazole** | 0.25 | 0.12 | 0.5 | 0.5 | 0.5 | 4 | 0.5 |
| **Itraconazole** | 0.25 | 0.25 | 0.5 | 0.5 | 0.5 | 1 | 0.5 |
| **Voriconazole** | 0.12 | 0.25 | 0.5 | 0.5 | 0.5 | 8 | 0.5 |
| **Caspofungin** | 0.5 | 0.5 | 8 | 8 | 8 | >16 | 8 |
