# Supplementary material for: Global Analysis of the Evolution and Mechanism of Echinocandin Resistance in Candida glabrata
Source: PLoS Pathog. 2012 May 17;8(5):e1002718. doi: 10.1371/journal.ppat.1002718 (PMC3355103; doi:10.1371/journal.ppat.1002718)
Supplement: Table S2 — Candida glabrata strains used in this study. (DOC) [file ppat.1002718.s004.doc]

**Table S2. *Candida glabrata* strains used in this study.**

| Strain Name | Genotype or phenotype | Source |
| --- | --- | --- |
| CgLC1461 | Prototrophic, clinical isolate A | This study |
| CgLC746 | Prototrophic, clinical isolate B | This study |
| CgLC747 | Prototrophic, clinical isolate C | This study |
| CgLC748 | Prototrophic, clinical isolate D | This study |
| CgLC749 | Prototrophic, clinical isolate E | This study |
| CgLC750 | Prototrophic, petite mutant, clinical isolate F | This study |
| CgLC751 | Prototrophic, clinical isolate G | This study |
| CgLC1272 | BG2, Prototrophic |  |
| CgLC1550 | Clinical isolate G *cnb1*::*HPH1* | This study |
| CgLC1551 | Clinical isolate G *cnb1*::*HPH1* | This study |
| CgLC1552 | Clinical isolate G *cnb1*::*HPH1* | This study |
| CgLC1910 | Clinical isolate A, respiration-deficient mutant isolated by EtBr treatment | This study |
| CgLC1911 | Clinical isolate A, respiration-deficient mutant isolated by EtBr treatment | This study |
| CgLC1912 | Clinical isolate A, respiration-deficient mutant isolated by EtBr treatment | This study |
| CgLC1913 | Clinical isolate G, respiration-deficient mutant isolated by EtBr treatment | This study |
| CgLC1914 | Clinical isolate G, respiration-deficient mutant isolated by EtBr treatment | This study |
| CgLC1915 | Clinical isolate G, respiration-deficient mutant isolated by EtBr treatment | This study |
| CgLC1916 | Clinical isolate G *cnb1*::*HPH1* respiration-deficient mutant isolated by EtBr treatment | This study |
| CgLC1917 | Clinical isolate G *cnb1*::*HPH1* respiration-deficient mutant isolated by EtBr treatment | This study |
| CgLC1918 | Clinical isolate G *cnb1*::*HPH1* respiration-deficient mutant isolated by EtBr treatment | This study |
| CgLC1943 | CAS08-0037, Clinical isolate harbouring *FKS2* T1987C (Fks2 S663P) |  |
| CgLC1944 | CAS08-0094, Clinical isolate harbouring *FKS2* T1987C (Fks2 S663P) |  |
| CgLC1945 | CAS08-0209, Clinical isolate harbouring *FKS2* T1987C (Fks2 S663P) |  |
| CgLC1946 | CAS08-0293, Clinical isolate harbouring *FKS2* T1987C (Fks2 S663P) |  |
| CgLC1947 | CAS08-0425, Clinical isolate harbouring *FKS2* T1987C (Fks2 S663P) |  |
| CgLC1948 | CAS09-0901, Clinical isolate harbouring *FKS2* T1987C (Fks2 S663P) |  |
| CgLC1949 | CAS09-1204, Clinical isolate harbouring *FKS2* T1987C (Fks2 S663P) |  |
| CgLC1950 | CAS09-1225, Clinical isolate harbouring *FKS2* T1987C (Fks2 S663P) |  |
| CgLC1951 | CAS09-1616, Clinical isolate harbouring *FKS2* T1987C (Fks2 S663P) |  |
| CgLC1952 | CAS09-1786, Clinical isolate harbouring *FKS2* T1987C (Fks2 S663P) |  |
| CgLC1998 | BG2, *FKS2* T1987C transformant #1 | This study |
| CgLC1999 | BG2, *FKS2* T1987C transformant #2 | This study |
| CgLC2000 | BG2, *FKS2* T1987C transformant #3 | This study |
| CgLC2001 | BG2, *FKS2* T1987C transformant #4 | This study |
| CgLC2121 | Clinical isolate G *HPH1*-*P_MET3_::HSP90* transformant #1 | This study |
| CgLC2122 | Clinical isolate G *HPH1*-*P_MET3_::HSP90* transformant #2 | This study |
| CgLC2151 | BG2 p*CDC6* WT (CgLC1272 + pLC650) | This study |
| CgLC2152 | BG2 p*CDC6* A511G (CgLC1272 + pLC651) | This study |
| CgLC2160 | BG2 *cdc6*::*HPH1* p*CDC6* WT (pLC650) transformant #1 | This study |
| CgLC2161 | BG2 *cdc6*::*HPH1* p*CDC6* WT (pLC650) transformant #2 | This study |
| CgLC2162 | BG2 *cdc6*::*HPH1* p*CDC6* A511G (pLC651) transformant #1 | This study |
| CgLC2163 | BG2 *cdc6*::*HPH1* p*CDC6* A511G (pLC651) transformant #2 | This study |
| CgLC2221 | BG2 *FKS2* T1987C p*CDC55* WT (pLC670) transformant #1 | This study |
| CgLC2222 | BG2 *FKS2* T1987C p*CDC55* WT (pLC671) transformant #2 | This study |
| CgLC2229 | BG2 *FKS2* T1987C p*CDC55* C463T (pLC672) transformant #1 | This study |
| CgLC2231 | BG2 *FKS2* T1987C p*CDC55* C463T (pLC673) transformant #2 | This study |
| CgLC2249 | BG2 *FKS2* T1987C pEmpty (pLC527) transformant #1 | This study |
| CgLC2250 | BG2 *FKS2* T1987C pEmpty (pLC527) transformant #2 | This study |
| CgLC2265 | BG2 p*CDC55* WT (pLC671) transformant #1 | This study |
| CgLC2266 | BG2 p*CDC55* WT (pLC671) transformant #2 | This study |
| CgLC2268 | BG2 p*CDC55* C463T (pLC672) transformant #1 | This study |
| CgLC2270 | BG2 p*CDC55* C463T (pLC673) transformant #2 | This study |
| CgLC2273 | BG2 pEmpty (pLC527) transformant #1 | This study |
| CgLC2274 | BG2 pEmpty (pLC527) transformant #2 | This study |
