# Supplementary material for: Global Analysis of the Evolution and Mechanism of Echinocandin Resistance in Candida glabrata
Source: PLoS Pathog. 2012 May 17;8(5):e1002718. doi: 10.1371/journal.ppat.1002718 (PMC3355103; doi:10.1371/journal.ppat.1002718)
Supplement: Table S3 — Plasmids used in this study. (DOC) [file ppat.1002718.s005.doc]

**Table S3. Plasmids used in this study.**

| Plasmid | Description | Source |
| --- | --- | --- |
| pLC527 | (pBM16) *S. cerevisiae* *TEF1p::NAT::CYC1*_3’UTR_ with *C. glabrata* *CEN/ARS*. Puc19-derived. |  |
| pLC530 | (pAP599) FRT-*pPGK1::Hph1*-FRT, ampR |  |
| pLC540 | *CgCNB1*-KO, ampR | This study |
| pLC650 | *CDC6* WT *NAT CEN/ARS* (derived from pLC527) | This study |
| pLC651 | *CDC6* A511G *NAT CEN/ARS* (derived from pLC527) | This study |
| pLC670/pLC671 | *CDC55* WT *NAT CEN/ARS* (derived from pLC527) | This study |
| pLC672/pLC673 | *CDC55* C463T *NAT CEN/ARS* (derived from pLC527) | This study |
