# Supplementary material for: Global Analysis of the Evolution and Mechanism of Echinocandin Resistance in Candida glabrata
Source: PLoS Pathog. 2012 May 17;8(5):e1002718. doi: 10.1371/journal.ppat.1002718 (PMC3355103; doi:10.1371/journal.ppat.1002718)
Supplement: Table S4 — Primers used in this study. (DOC) [file ppat.1002718.s006.doc]

**Table S4. Primers used in this study.**

| Primer | Description | Sequence |
| --- | --- | --- |
| oLC243 | M13Rev | CAGGAAACAGCTATGAC |
| oLC244 | M13Fwd | GTAAAACGACGGCCAG |
| oLC1208 | CgCNB1-346F-SacI | CCCGAGCTCAGTTCCTTACCTAGTGCAGC |
| oLC1209 | CgCNB1+3R-NotI | ATAAGAATGCGGCCGCCATTTCCCTTGATTAATACT |
| oLC1210 | CgCNB1+525F-SalI | ACGTGTCGACTAGTTCAGGCAAGAAACTTAACG |
| oLC1211 | CgCNB1+888R-KpnI | CGGGGTACCGCATTTATATTGTGCCAACC |
| oLC1212 | CgCNB1-509F | GGTCAAACTGTATGTGATGC |
| oLC1213 | CgCNB1+1104R | ATGAAAGGGCGATGATAACG |
| oLC1214 | CgCNB1+23F | TGTAGAGACTCTATTGGAGG |
| oLC1215 | CgCNB1+508R | TCAGGCTCTTTGCTACTTCG |
| oLC1216 | pAP599R | AGGAAGACAGGAAGTCGAGC |
| oLC1217 | pAP599F | GCACGAACACGATATAGAGG |
| oLC1344 | CgFKS2+1651F | AGGTTTTGGTTTTTATGTGGTATCC |
| oLC1345 | CgFKS2+2225R | ATATTTCTCCAAGGAGTTAAGATGG |
| oLC1388 | Hph+600R | GAAATCCGCGTGCACGAGGT |
| oLC1389 | Hph+377F | CCGATCTTAGCCAGACGAGC |
| oLC1400 | CgHSP90-667F-SacI | CCCGAGCTCCGTTCTGCGCAGAAACTTCC |
| oLC1401 | CgHSP90-250R-NotI | ATAAGAATGCGGCCGCAAAGAACGCAACACGCCAACAGC |
| oLC1406 | CgHSP90-781F | AAGAGATACGAACATCAAGG |
| oLC1407 | CgHSP90+519R | TCTTAGGATGGTACCTCTGC |
| oLC1498 | CgFKS2+4819F | ACCGGTGTTAAGGCCACCGA |
| oLC1499 | CgFKS2+4997R | ACCGCCGCCATAACAGCACC |
| oLC1500 | CgACT1+481F | TTGAGAATCGACCTTGCTGG |
| oLC1501 | CgACT1+637R | GCATTTCTTGTTCGAAGTCC |
| oLC1502 | CgCNB1+142F | AGTATACCTGGCGTGTCTGC |
| oLC1503 | CgCNB1+337R | CGTTGGATATCAGACCATCC |
| oLC1559 | CgDOT6+895F | TCCACGCAGTCTATTACAGG |
| oLC1560 | CgDOT6+1401R | AGAAATGTTGAATGACGACC |
| oLC1561 | CgMOH1-187F | CTACCTGGACGTTTGGATGC |
| oLC1562 | CgMOH1+320R | TCCATTAAGTACGCTGTTCC |
| oLC1563 | CgGPH1+1032F | GGAATTGAGATTGAAGCAGC |
| oLC1564 | CgGPH1+1614R | TCTTGGTGTGATACCATTGG |
| oLC1565 | CgMRPL11+164F | CGCATTACAACAACTTGACG |
| oLC1566 | CgMRPL11+642R | ATCTTCGTGGGATTTCAAGG |
| oLC1567 | CgCDC6+358F | TCGCTGTACATAACAGGTCC |
| oLC1568 | CgCDC6+916R | CAGTATATGGTTGAAAGACC |
| oLC1569 | CgCDC55+291F | AAATAAGTCGCACTTCTTGC |
| oLC1570 | CgCDC55+835R | TGTGATTCATTGGATCCAGG |
| oLC1571 | CgSUI2+193F | GTTGGTAAGAACGATGTTGC |
| oLC1572 | CgSUI2+711R | CTTGTCTAGAGCTTGAGTGG |
| oLC1573 | CgTCB1+3118F | TACAATGATGAAGACGATGC |
| oLC1574 | CgTCB1+3591R | TCAATCATCTTCGTTATGC |
| oLC1792 | pLC527+1447F | TACCGCACAGATGCGTAAGG |
| oLC1793 | pLC527+1821R | GAGTTAGCTCACTCATTAGG |
| oLC1900 | CgFKS2+1942F G1980A T1987C | GTTTTTGCTGCCAAGTATTCTGAATCGTACTTCTTCTTaATTTTGCCTCTAAGAGACCCTATCAGAATTTTATCAACTACTACCATGAGA |
| oLC1980 | CgMET3-1026F-ClaI | ACGTATCGATATACCAGTTACAATTAGTAT |
| oLC1981 | CgpMET3-1-CgHSP90+26R | GCTTGAAATTCAAAAGTTTCAGACATttgttaggtgtttcttttctgg |
| oLC1982 | CgpMET3-1-CgHSP90+26F | ccagaaaagaaacacctaacaaATGTCTGAAACTTTTGAATTTCAAGC |
| oLC1983 | CgHSP90+407R-SalI | ACGTgtcgacATAACTTGGACTCTGTCAGC |
| oLC1984 | CgpMET3-219F | TTATAACTGTGCATCTTGCC |
| oLC1985 | CgpMET3-352F | GATCCTTCGCTAGCATATCC |
| oLC2003 | CgCDC6-513F-SacI | CCC**GAGCTC**ATACCAGGGAAGTGAGAACC |
| oLC2004 | CgCDC6+1848R-KpnI | CGG**GGTACC**TGCTCCGCAATATAAGCTCG |
| oLC2171 | CgCDC6-513F | ATACCAGGGAAGTGAGAACC |
| oLC2176 | CgCDC6+1848R | TGCTCCGCAATATAAGCTCG |
| oLC2181 | pLC530+2123R-CgCDC6+3R | CGAGCGACGGTAATTTCTTTCTCATCTCGGTTTGTTGTTTGTTTTGACG |
| oLC2182 | CgCDC6-27F-pLC530-2120F | CGTCAAAACAAACAACAAACCGAGATGAGAAAGAAATTACCGTCGCTCG |
| oLC2183 | CgCDC6+1623R-pLC530+4099R | CTCAAGCAATACCATATTGCGATATCGAATTCCTGCAgCC |
| oLC2184 | pLC530+4080F-CgCDC6+1604F | GGcTGCAGGAATTCGATATCGCAATATGGTATTGCTTGAG |
| oLC2187 | CgCDC6-632F | CGAAGGAAGAAATCAGATGG |
| oLC2188 | CgCDC6+1931R | CTTGATAGTGGTGATTTTCC |
| oLC2229 | CgCDC55-505F | ACTACTTATCTACCTATTCC |
| oLC2234 | CgCDC55+2023R | TAAATCTTACCATAACCAGG |
| oLC2237 | CgCDC55-505F-SacI | TTGCGAGCTCACTACTTATCTACCTATTCC |
| oLC2238 | CgCDC55+2163R-SalI | ACGTGTCGACTAAATCTTACCATAACCAGG |
| oLC2239 | CgCDC55+3R | CATTCTTTCTTTCTCTTTTGC |
| oLC2240 | CgCDC55+1486F | TAACTTTTATAGCTTTGTTAGG |
| oLC2247 | CgFKS2+1942F G1980A | GTTTTTGCTGCCAAGTATTCTGAATCGTACTTCTTCTTaATTTTGTCTCTAAGAGACCCTATCAGAATTTTATCAACTACTACCATGAGA |
