# Supplementary material for: Global Analysis of the Evolution and Mechanism of Echinocandin Resistance in Candida glabrata
Source: PLoS Pathog. 2012 May 17;8(5):e1002718. doi: 10.1371/journal.ppat.1002718 (PMC3355103; doi:10.1371/journal.ppat.1002718)
Supplement: Text S1 — Supporting Materials and Methods . (DOC) [file ppat.1002718.s007.doc]

**Text S1**

**Materials and Methods**

***Strain construction***

**CgLC1550, 1551, 1552**

To knock-out *CgCNB1* in *C. glabrata*, a split-marker PCR method using pLC540 was employed. pLC540 contains ~400bp of DNA sequence homologous to *CgCNB1* flanking an *HPH1* cassette with FRT sites for recycling of the marker. Primers oLC1208/1388 were used to PCR amplify the first half of this cassette (1581 bp) and oLC1389/1211 were used to PCR amplify the second half of the cassette. The first and second halves have 200bp of overlapping sequence in the middle of Hph1 such that transformation with equal amounts of the two halves requires three separate recombination events to get hygromycin B resistance, reducing the amount of illegitimate recombination. 20 µg of DNA was used in the transformation, cells were harvested for standard LiOAC transformation at OD600 = 0.4, with 10% DMSO added right before heat shock. Upstream integration was confirmed by PCR using primers oLC1212/1216 (690 bp) and downstream integration was confirmed by PCR using oLC1213/1217 (685 bp). Lack of a wild-type allele was confirmed by PCR with oLC1214/1215 (485 bp if a wild-type allele is still present). Three separate transformants were archived and used in subsequent experiments.

**CgLC1998, 1999, 2000, 2001**

The *CgFKS2* T1987C mutation was introduced in CgLC1272 by transformation via lithium acetate with 10 µg of the synthetic oligonucleotide oLC1900, a 90-mer containing the T1987C mutation in the center as well as a silent mutation G1980A to track resistant mutants that arise due to homologous recombination or oLC2247, a control 90-mer containing no *FKS2* mutation except for the silent tracker mutation. Transformants were selected for on YPD agar with 1 µg/ml caspofungin. The *CgFKS2* hot spot 1 region of the transformants was PCR amplified with oLC1344/1345 and sequenced with oLC1344 to verify both the presence of the *CgFKS2* T1987C mutation as well as the silent G1980A mutation to differentiate between homologous recombinants versus spontaneous mutants. Four sequence-verified transformants were archived and used in subsequent experiments.

**CgLC2121, CgLC2122**

*HSP90* was promoter-replaced with the *MET3* promoter in late clinical isolate G (CgLC751). Using the split-marker approach, the upstream half of the *HPH1*- *PMET3::HSP90* cassette from pLC658 was PCR amplified with oLC1388/1400 (1649 bp) and the downstream half of the cassette with oLC1389/1983 (2573 bp). 10 µl of each half was transformed into CgLC751 and strains were outgrown in SD media lacking methionine and cysteine for 6 hours and selected on SD + HygB (250 µg/ml). Transformants were PCR tested with oLC1406/1216 (706 bp) and oLC1984/1407 (738 bp). Positive transformants were also tested for responsiveness (lack of growth) on SD medium supplemented with methionine.

**CgLC2151**

*CDC6* isolated from clinical isolate A was ectopically expressed in *C. glabrata* laboratory strain BG2 (CgLC1272). CgLC1272 was transformed via lithium acetate with pLC650 and NAT-resistant transformants were verified by PCR for presence of the plasmid with oLC1792/1568 (1638 bp). The plasmid was maintained with 150 µg/ml NAT.

**CgLC2152**

*CDC6* isolated from clinical isolate G was ectopically expressed in *C. glabrata* laboratory strain BG2 (CgLC1272). CgLC1272 was transformed via lithium acetate with pLC651 and NAT-resistant transformants were verified by PCR for presence of the plasmid with oLC1792/1568 (1638 bp). The plasmid was maintained with 150 µg/ml NAT.

**CgLC2160, CgLC2161**

Genomic *CDC6* was knocked out of CgLC2151 using a split-marker fusion PCR method so that the only allele of *CDC6* expressed is the plasmid-borne copy (pLC650). ~500 bp of upstream homology of *CDC6* before the start codon was amplified from genomic DNA with oLC2171/2181 and the first half of the hygromycin B resistance cassette was amplified from pLC530 with oLC2182/1388. The PCR products were fused with oLC2171/1388 to create an upstream knockout cassette. Downstream homology of *CDC6* after the stop codon was amplified from genomic DNA with oLC2184/2176 and the second half of the hygromycin B resistance cassette, including a 200bp overlapping region with the first half, was amplified from pLC530 with oLC1389/2183. The PCR products were fused with oLC1389/2176 to create a downstream knoc-out cassette. CgLC2151 was transformed via lithium acetate with 10 µl of each of the upstream and downstream knockout cassettes. Transformants were selected on YPD + NAT + Hygromycin B in order to maintain plasmid selection and were PCR tested for upstream integration of the knockout cassette with oLC2187/1216 (717 bp), downstream integration with oLC1217/2188 (408 bp), presence of the plasmid-derived *CDC6* with oLC1792/1568 (1638 bp) and lack of a duplicated wild-type allele with oLC2187/1568 (1548 bp). Two transformants were archived.

**CgLC2162, CgLC2163**

Created as above except the parent strain is CgLC2152.

**CgLC2221**

CgLC1998 was transformed via lithium acetate with pLC670. NAT-resistant transformants were PCR tested with oLC1792/2239 (717 bp) for presence of the plasmid. The plasmid was maintained with 150 µg/ml NAT.

**CgLC2222**

CgLC1998 was transformed via lithium acetate with pLC671. NAT-resistant transformants were PCR tested with oLC1792/2239 (717 bp) for presence of the plasmid. The plasmid was maintained with 150 µg/ml NAT.

**CgLC2229**

CgLC1998 was transformed via lithium acetate with pLC672. NAT-resistant transformants were PCR tested with oLC1792/2239 (717 bp) for presence of the plasmid. The plasmid was maintained with 150 µg/ml NAT.

**CgLC2231**

CgLC1998 was transformed via lithium acetate with pLC673. NAT-resistant transformants were PCR tested with oLC1792/2239 (717 bp) for presence of the plasmid. The plasmid was maintained with 150 µg/ml NAT.

**CgLC2249, CgLC2250**

CgLC1998 was transformed via lithium acetate with pLC527 to serve as an empty vector control. NAT-resistant transformants were PCR tested with oLC1792/1793 (375 bp) for presence of the plasmid. The plasmid was maintained with 150 µg/ml NAT. Two transformants were archived.

**CgLC2265, CgLC2266**

CgLC1272 was transformed via lithium acetate with pLC671. NAT-resistant transformants were PCR tested with oLC1792/2239 (717 bp) for presence of the plasmid. The plasmid was maintained with 150 µg/ml NAT. Two transformants were archived.

**CgLC2268**

CgLC1272 was transformed via lithium acetate with pLC672. NAT-resistant transformants were PCR tested with oLC1792/2239 (717 bp) for presence of the plasmid. The plasmid was maintained with 150 µg/ml NAT.

**CgLC2270**

CgLC1272 was transformed via lithium acetate with pLC673. NAT-resistant transformants were PCR tested with oLC1792/2239 (717 bp) for presence of the plasmid. The plasmid was maintained with 150 µg/ml NAT.

**CgLC2273, CgLC2274**

CgLC1272 was transformed via lithium acetate with pLC527 to serve as an empty vector control. NAT-resistant transformants were PCR tested with oLC1792/1793 (375 bp) for presence of the plasmid. The plasmid was maintained with 150 µg/ml NAT. Two transformants were archived.

***Plasmid construction***

**pLC540**

DNA sequence homologous to upstream of *CgCNB1* was PCR amplified from genomic DNA with oLC1208/oLC1209 and cloned into pLC530 at *Sac*I and *Not*I. Sequence homologous to downstream of *CgCNB1* was PCR amplified from genomic DNA with primers oLC1210/oLC1211 and cloned into pLC530 containing the upstream homology at *Sal*I and *Kpn*I. The presence of the inserts was tested by PCR with oLC1208/oLC1216 and oLC1211/oLC1217.

**pLC650**

Wild-type *CDC6* was amplified from isolate A (CgLC1461) genomic DNA with oLC2003/2004 (2373 bp) and cloned into pLC527 at *Kpn*I/*Sac*I. Integration was confirmed via PCR with oLC1792/1568 (1638 bp) and oLC1567/1793 (1657 bp). The sequence of *CDC6* was verified with oLC243, oLC244, and oLC1567.

**pLC651**

Constructed and verified as with pLC650 except *CDC6* was amplified from isolate G (CgLC751) genomic DNA.

**pLC658**

Upstream homology of *CgHSP90* was amplified from genomic DNA with oLC1400/1401 (431 bp) and cloned into the *Sac*I/*Not*I sites in pLC530. Integration was tested with oLC1216/1400 (601bp). *CgpMET3* was amplified from genomic DNA with oLC1980/1981 (1058 bp) and *CgHSP90* downstream homology was amplified from genomic DNA with oLC1982/1983 (435 bp). PCR products were fused via PCR with oLC1980/1983 and the fusion product was gel purified, and cloned into *Cla*I/*Sal*I in pLC530 containing the upstream homology region of *CgHSP90*. Correct integration was tested with oLC243/1984 (721 bp) and oLC1217/1985 (772 bp). For the split-marker approach, amplify the upstream half of the cassette with oLC1388/1400 (1649 bp) and the downstream half of the cassette with oLC1389/1983 (2573 bp). Transform with equal amounts of each PCR product.

**pLC670, pLC671**

*CDC55* was amplified from isolate A (CgLC1461) genomic DNA with oLC2237/2238 (2540 bp) and cloned into pLC527 at *Sal*I/*Sac*I. Integration was confirmed via PCR with oLC1793/2240 (683 bp). The sequence of *CDC55* was verified with oLC2229, oLC1569, and oLC2234. Two independent plasmids with the identical *CDC55* sequence were archived.

**pLC672, pLC673**

*CDC55*was amplified from isolate G (CgLC751) genomic DNA with oLC2237/2238 (2540 bp) and cloned into pLC527 at *Sal*I/*Sac*I. Integration was confirmed via PCR with oLC1793/2240 (683 bp). The sequence of *CDC55* was verified with oLC2229, oLC1569, and oLC2234. Two independent plasmids with the identical *CDC55* sequence were archived.

**Supplemental References**

1. Fidel PL, Jr., Cutright JL, Tait L, Sobel JD (1996) A murine model of *Candida glabrata* vaginitis. Journal Infect Dis 173: 425-431.

2. Zimbeck AJ, Iqbal N, Ahlquist AM, Farley MM, Harrison LH, et al. (2010) *FKS* mutations and elevated echinocandin MIC values among *Candida glabrata* isolates from U.S. population-based surveillance. Antimicrob Agents Chemother 54: 5042-5047.

3. Ma B, Pan SJ, Domergue R, Rigby T, Whiteway M, et al. (2009) High-affinity transporters for NAD+ precursors in *Candida glabrata* are regulated by Hst1 and induced in response to niacin limitation. Mol Cell Biol 29: 4067-4079.

4. Domergue R, Castano I, De Las Penas A, Zupancic M, Lockatell V, et al. (2005) Nicotinic acid limitation regulates silencing of *Candida* adhesins during UTI. Science 308: 866-870.
